# A Hybrid Positioning Framework for Large-Scale Three-Dimensional IoT Environments

**DOI:** 10.3390/s25226943

**Published:** 2025-11-13

**Authors:** Shima Koulaeizadeh, Hatef Javadi, Sudabeh Gholizadeh, Saeid Barshandeh, Giuseppe Loseto, Nicola Epicoco

**Affiliations:** 1 Communication Networks, Technische Universität, 09111 Chemnitz, Germany; shima.koulaeizadeh@s2024.tu-chemnitz.de; 2 Department of Industrial Engineering, Hacettepe University, Ankara 06800, Türkiye; hatef.javadi@hacettepe.edu.tr; 3 Department of Computer Science, School of Engineering, Afagh Higher Education Institute, Urmia 57158-55700, Iran; sudabeh.gholizadeh7070@gmail.com; 4 Department of Engineering, LUM University, 70010 Casamassima, Italy; loseto@lum.it (G.L.); epicoco@lum.it (N.E.)

**Keywords:** three-dimensional Internet of Things, distance vector-hop, positioning, localization, optimization

## Abstract

The Internet of Things (IoT) and Edge Computing (EC) play an essential role in today’s communication systems, supporting diverse applications in industry, healthcare, and environmental monitoring; however, these technologies face a major challenge in accurately determining the geographic origin of sensed data, as such data are meaningful only when their source location is known. The use of Global Positioning System (GPS) is often impractical or inefficient in many environments due to limited satellite coverage, high energy consumption, and environmental interference. This paper recruits the Distance Vector-Hop (DV-Hop), Jellyfish Search (JS), and Artificial Rabbits Optimization (ARO) algorithms and presents an innovative GPS-free positioning framework for three-dimensional (3D) EC environments. In the proposed framework, the basic DV-Hop and multi-angulation algorithms are generalized for three-dimensional environments. Next, both algorithms are structurally modified and integrated in a complementary manner to balance exploration and exploitation. Furthermore, a Lévy flight-based perturbation phase and a local search mechanism are incorporated to enhance convergence speed and solution precision. To evaluate performance, sixteen 3D IoT environments with different configurations were simulated, and the results were compared with nine state-of-the-art localization algorithms using MSE, NLE, ALE, and LEV metrics. The quantitative relative improvement ratio test demonstrates that the proposed method is, on average, 39% more accurate than its competitors.

## 1. Introduction

Edge Computing (EC) is the most contemporary communication paradigm in today’s networks, employed in numerous fields [1]. The Internet of Things (IoT) forms the bottommost layer of EC environments, which is also called the edge layer. This layer generally comprises end-user devices, sensors, actuators, cameras, etc. In data-oriented surveillance networks, the devices of this layer collect environmental data and send them to the base stations/sinks or higher layers for further processing [2]. However, this layer’s sensors, actuators, and other equipment face the processing, storage, communication, and especially energy resource constraints [3]. Interest in the field of location-aware services in IoT, in which the geographical location of devices and their sensed data from the surroundings is crucial, is growing enormously. Detecting the location of IoT devices and their data is one of the fundamental requirements of IoT and EC networks for many wireless applications, which is known as localization/positioning [4].

Locating data, sensors, and wireless IoT devices is a challenging problem due to the dynamic and erratic nature of environments and the limited resources of devices, particularly energy limitations. Existing traditional approaches, including GPS-based methods, manual measurement methods, etc., are not appropriate for today’s dynamic large-scale networks with high data volume due to reasons such as high cost of procurement and maintenance, lack of re-access to devices, limited energy, coverage constraints, and high time consumption. Consequently, along with the development of network equipment and communication technologies, the demand for optimal positioning algorithms is felt more and more [5].

Utilizing GPS can be practical, but the accuracy of its position estimation in indoor environments, deep forests, under the sea, etc., is not satisfactory enough. Also, embedding GPS in surveillance equipment increases the cost of implementation and setup [6]. Additionally, the GPS hardware increases power consumption, which is inappropriate for power-constrained devices. As a result, researchers conducted various studies to devise alternative methods aimed at how to use the interaction and connectivity between nodes to estimate position [7].

The need for GPS-free localization arises prominently in environments where satellite signals are unavailable, attenuated, or unreliable. For instance, in underground mines, tunnels, and subterranean infrastructures, GPS signals cannot penetrate soil or rock layers, making traditional satellite-based localization infeasible. In such contexts, accurate positioning of IoT-enabled equipment, robots, and personnel faces major challenges. Similarly, disaster zones, such as collapsed buildings, forest fires, or earthquake-affected areas, often experience severe signal blockage or infrastructure damage. Deploying GPS-free IoT localization systems in these scenarios allows first responders to accurately locate trapped victims, assess structural integrity, and coordinate rescue operations in real time without relying on external satellite connectivity [8].

Furthermore, industrial and urban environments—including smart factories, large warehouses, oil refineries, and indoor logistics facilities—frequently suffer from multipath fading, electromagnetic interference, and signal occlusion caused by dense metallic structures and machinery. In these harsh or cluttered environments, GPS-based positioning can become highly inaccurate or entirely unavailable. Consequently, GPS-free localization frameworks provide a reliable alternative for maintaining continuous and precise positioning. They enable efficient asset tracking, autonomous robotic operations, and process monitoring even under severe signal distortions, thereby ensuring robust and energy-efficient operation of IoT and edge computing networks in real-world industrial and emergency scenarios [9].

The GPS-free positioning strategies are generally classified into range-based and range-free algorithms. Range-based localization algorithms require additional equipment to acquire some necessary information, such as signal strength or received angle, to estimate the position of unknown devices [10]. These methods have higher accuracy, but, due to the need for additional equipment, they are more costly. In contrast, range-free algorithms estimate the location of unknown devices by the nature of the network, the relationship between devices, the connectivity and density of the network, the number of hops, and distance calculation. Range-free algorithms have relatively lower positioning accuracy, but, due to their low cost and simplicity, they are more suitable for today’s large-scale networks [11].

Among the range-free positioning algorithms, the Distance Vector-Hop (DV-Hop) is the most optimal, simple, and widely used method for estimating the position of IoT devices in large-scale networks [12]. The DV-Hop estimates the distance through the number of hops between unknown IoT nodes and beacons. The DV-Hop returns the same distances for the nodes whose hop counts from the beacon are identical. This is due to DV-Hop’s use of the average step size, while the length of the hops in the network is disparate. Therefore, the DV-Hop estimates positions with a major error, especially in three-dimensional, large-scale environments. Accordingly, researchers have attempted in recent years to enhance DV-Hop’s accuracy by embedding other techniques in it [13].

In the hybrid methods, the combination of DV-Hop with meta-heuristic algorithms has been highly regarded, and the resulting algorithms indicated adequate performance and accuracy [14]. Metaheuristic algorithms do not demand any information about the problem [15]. As a result, these algorithms are applied to a wide range of complex real-world problems. These algorithms are also able to find a near-optimal solution for the given problem with any set of initial solutions [16]. The optimality of the final solution obtained by them can be adjusted by two parameters: the number of iterations and the number of search agents. The higher the values of these parameters are, the more optimal the final solution will be, but the execution time will also increase [17].

In this research, a new positioning algorithm named EJSARO is presented for 3D IoT networks based on the hybridization of the DV-Hop and meta-heuristic algorithms. In the first step of the proposed method, the basic VD-Hop algorithm has been extended to count hops and estimate the distance in three-dimensional environments. Next, the multi-literation method is recruited to estimate the initial positions of unknown objects using the distance and hops calculated by 3D-DV-Hop. Then, a new hybrid metaheuristic algorithm is introduced based on Jellyfish Search (JS) and Artificial Rabbits Optimization (ARO) algorithms to improve the initially estimated positions. In the proposed hybrid metaheuristic algorithm, the original ARO and JS algorithms are modified and lightened. The modified JS and ARO are then hybridized in a complementary manner. Subsequently, the outcome hybrid algorithm is enhanced by the Lévy flight function-based intermediate phase. Additionally, a new local search mechanism is provided to exploit the best area discovered by the algorithm.

To put it concisely, the main contributions and novelties of our work are as below:

- The classical DV-Hop algorithm is mathematically generalized from 2D to 3D environments, redefining hop-count computation, distance estimation, and multilateration equations.
- A new hybrid optimization algorithm combining Jellyfish Search (JS) and Artificial Rabbits Optimization (ARO) is proposed.
- Both algorithms are structurally modified: redundant mechanisms are removed, and key operators are redesigned to achieve a lighter and faster hybrid while preserving exploration–exploitation balance.
- The hybridization is complementary, not sequential—each component enhances the other’s deficiencies, which has not been performed before.
- A Lévy flight-driven motion phase is introduced to enable controlled random jumps, enhancing the ability to escape local optima and explore the search space globally.
- A counter-based local departure mechanism is introduced to detect stagnation and dynamically reinitialize stuck solutions, ensuring diversity and maintaining convergence stability. This mechanism acts as an adaptive restart process—a unique feature not seen in previous hybrid positioning algorithms.
- A greedy local search strategy is provided to improve the best-found solution iteratively by exploiting its neighborhood, increasing the precision of final position estimates.
- Sixteen diverse 3D IoT environments were designed to test the performance of the positioning methods under varying densities, scales, and anchor configurations—something rarely seen in prior 3D localization works.
- The proposed framework is evaluated against nine state-of-the-art algorithms, showing superior Mean Square Error (MSE), Node Localization Error (NLE), Average Localization Error (ALE), and Localization Error Variance (LEV) performance metrics.

The rest of this paper is organized as follows: Section 2 explores related research. Section 3 introduces the basic algorithms employed in developing the proposed algorithm. Section 4 exposes the implementation details of the proposed method and the contributions made. Section 5 provides the results of extensive experiments conducted to assess the potency of the proposed method. Eventually, Section 6 summarizes this paper and gives directions for future research.

## 2. Literature Review

Various studies have been conducted in the field of refining data aggregation, improving quality-of-service (QoS), reducing communication time, faster computing, achieving real-time communication, and diminishing energy consumption to augment IoT networks’ productivity. The positioning/localization problem is one of the critical challenges faced by IoT networks because, in most applications, these networks require location information. Accordingly, numerous research studies have been conducted in this orientation. For instance, Yang and Wang employed the Grey Wolf Optimizer and DV-hop algorithm and presented a new localization algorithm for WSN-based IoT networks [18]. In the proposed method, the hop counts obtained by the DV-Hop algorithm are refined by a dual communication radius. The authors claim that using a dual communication radius can enhance the hop count estimation process. In the next step, the minimum hop count between beacons is obtained by a new hop adjustment factor, which can be calculated from the average hop distance more accurately. Likewise, the hop distances between unknown IoT devices and the beacons are estimated through a weighted optimization regarding the mean square error criterion. Then, the basic GWO is improved by some modifications and used to compute the position of unknown nodes. The experimental results show that the proposed algorithm has lower localization.

Similarly, Sun and Yang employed the Black-winged Kite Algorithm (BKA) and presented a range-free positioning model for two-dimensional WSN environments [19]. In the proposed algorithm, the accuracy of the distance estimation obtained by the DV-Hop algorithm is enhanced through a new error factor. This factor refines the average hop distance calculation and reduces cumulative errors in node localization. Next, the original BKA is improved by incorporating Gaussian mutation, opposition-based learning, and an optimal individual shake strategy. These modifications augment the local optima avoidance and accelerate the convergence rate. The results of the proposed algorithm are compared with similar methods, comprising ICSFG, TSHH, RANSAC, and IAGA models. The comparison results showed that the proposed algorithm performed better than competitors in wireless sensor network localization.

Likewise, Al Janabi and Kurnaz presented a novel localization mechanism for IoT networks by integrating the Grasshopper Optimization Algorithm (GOA) with the DV-Hop algorithm [11]. The main goal of the authors in this paper was to improve localization accuracy in wireless sensor networks. In the proposed method, the hops between unknown nodes and three beacons are obtained to address scenarios with fewer than three GPS-enabled beacons. In the following, the position of objects is estimated according to the hops and calculated distances from those beacons. After that, the GOA is used to optimize the estimated positions by generating candidate solutions around the positions computed by the DV-Hop with the aim of minimizing localization error. In this regard, each position vector is considered a grasshopper in the swarm. The proposed algorithm is compared with algorithms like PSO, Firefly, and the Butterfly Optimization algorithm, and the results indicate a lower localization error.

Moreover, Singh et al. recruited the Reptile Search Algorithm and Locally Linear Embedding (LLE) algorithm and provided a new localization framework for WSN-assisted IoT networks [20]. In the proposed framework, the RSA optimizes node positions iteratively to minimize localization error in noisy/dynamic WSN environments. Then, the LLE, a manifold learning technique, reduces high-dimensional sensor data into a lower-dimensional space while preserving local node relationships. Simulation results demonstrate a reduction in localization error and computational complexity for varying sensor nodes and fixed anchor nodes. Also, the results indicate lower energy consumption in different scenarios.

In [4], Barshandeh et al. presented a new positioning system for locating objects in 3D Internet of Things networks. The proposed method is based on the Received Signal Strength Indicator (RSSI) algorithm, Slime Mold Algorithm (SMA), and Equilibrium Optimizer (EO). In the first step of the proposed algorithm, the initial position of the IoT devices is estimated by the RSSI algorithm. Then, the accuracy of the initial estimated locations is improved by the hybrid optimization algorithm. In the hybrid algorithm, the SMA algorithm is corrected and then combined with the EO algorithm through a learning mechanism. In each iteration of the algorithm, the learning strategy specifies which equation updates the solution. Additionally, a neighborhood search strategy is presented to improve the search process. To evaluate the merit of the proposed algorithm, the results obtained on fifteen 3D Internet of Things networks have been compared with the AEO, AO, EO, MRFO, SMA, WOA, PSO, and SSA algorithms. The experimental results indicate the superiority of the proposed method over competing algorithms.

In a similar research, Barshandeh et al. presented a novel localization method for two-dimensional IoT networks using the DV-Hop [5]. In this method, after obtaining the initial location of unknown network nodes, the accuracy of the initially detected locations is improved by a hybrid meta-heuristic algorithm. In the proposed hybrid meta-heuristic algorithm, the Tunicate Swarm Algorithm (TSA) is combined with Harris Hawks Optimization (HHO). Next, the resulting hybrid algorithm is improved by a new phase. The experiments conducted in this research are divided into two parts. In the first part, the efficiency of the improved hybrid optimization algorithm is evaluated using 50 benchmark functions. In the second part, the efficiency of the proposed positioning algorithm has been tested on twenty-eight Internet of Things networks. The obtained results of the proposed method are compared with the HHO, TSA, GWO, SCA, WOA, and CSA algorithms using node location error, average location error, and location error variance measures.

Also, in [21], Liu et al. presented a new localization algorithm called OTKL-BSA to estimate the location of nodes in underwater wireless sensor networks. In the OTKL-BSA, the underwater nodes are divided into two levels. Next, the clock asynchronous localization system (LS-AC) algorithm estimates the position of base layer nodes. To remove the dependence of the range-based strategy on active nodes and address the energy consumption issue, LS-AC performs state-based monitoring within the network by relying on asynchronous clocks. Also, using optimal topology and knowledge learning, a backtracking search algorithm (BSA) is introduced in this paper. The BSA has been used to eliminate the lack of dispersion of solutions and the imbalance of exploration and exploitation capabilities. In addition, the Gray Wolf Optimizer (GWO) algorithm was improved in this study by a hunting step size mechanism. To evaluate the efficiency of the OTKL-BS method, its results are compared with SLMP, MCL-MP, MP-PSO, and MGP.

Moreover, Soundararajan et al. have proposed a meta-heuristic-based positioning algorithm and a multi-hop routing method called MONL-MRPMS for Wireless Sensor Networks (WSN) [22]. The main goal of the authors in this research was to optimize energy consumption in WSN networks with a mobile sink by accurately locating network nodes. In the MONL-MRPMS algorithm, the Coyote Optimization Algorithm (COA), in which the Euclidean distance is considered as the fitness function, is used for the positioning of unknown nodes. Additionally, the Seagull Optimization Algorithm (SGO) has been used in the MONL-MRPMS to select the most optimal multi-hop routes between clusters. In the proposed method, a mobile sink is also considered, which optimizes the energy consumption in the network by adjusting the routes when the sink moves.

Additionally, Fute et al. recruited Particle Swarm Optimization (PSO) and Tabu Search (TS) and presented a hybrid positioning algorithm called FPSOTS for wireless sensor networks [23]. In FPSOTS, the PSO algorithm is improved by the strategies of the TS. In this regard, each particle in PSO uses TS to determine its best neighbor. By selecting the best neighbors, the convergence rate of the solutions towards the global optimum can be increased. In addition, the limit and performance checking procedures are added to the PSO so that only the best solutions contribute to the update of other solutions. In the FPSOTS, the initial position of unknown nodes is estimated by the received signal strength indicator (RSSI) and trilateration algorithms. In the experimental section, the results of the proposed method have been compared with the HPSOVNS, NS-IPSO, ECS-NL, and GTOA algorithms in terms of localization accuracy and convergence criteria.

Additionally, in [24], Cao et al. presented a hybrid swarm intelligence-based localization algorithm for Non-Line-of-Sight (NLoS) environments. Their proposed method recruits the Time Difference of Arrival (TDOA) and Angle of Arrival (AOA) algorithms. The TDOA and AOA are improved in this paper by the Crow Search Algorithm (CSA) and Particle Swarm Optimization (PSO). In this regard, the hybrid CSA and PSO algorithm solves the non-linear equations of the TDOA and AOA algorithms. Also, to obtain a better fitness value during the optimization process and increase the localization accuracy, the fitness function is modified based on maximum likelihood estimation. Then, an initial solution is periodically added to the population to increase the convergence of the algorithm and optimize the global search, causing a reduction in the diversity of the population. The authors have compared the results of their proposed method with the results of the Taylor, Chan, PSO, CPSO, and CSA algorithms.

Despite the progress made by existing metaheuristic-based localization algorithms, several research gaps remain unaddressed. Most current methods—such as GWO-based, SMA-based, or other single-optimizer frameworks—tend to exhibit premature convergence and limited adaptability when applied to large-scale or three-dimensional IoT environments. These algorithms, while fast in convergence, often lose population diversity during the later search phases, leading to suboptimal accuracy. While effective for exploration, these approaches often converge unstably and exploit local areas weakly, leading to inconsistent performance in diverse network conditions. Furthermore, many hybrid or improved versions of these algorithms increase computational complexity without providing consistent accuracy gains. These shortcomings highlight the need for a balanced and computationally efficient hybrid optimization strategy that can maintain diversity, avoid local stagnation, and refine position estimates precisely. To address these limitations, this study proposes the EJSARO hybrid framework, which synergistically integrates the complementary strengths of Artificial Rabbits Optimization (ARO) and Jellyfish Search (JS), augmented with Lévy-flight perturbation and a local search mechanism to enhance convergence stability and positioning accuracy in 3D IoT environments.

The above-mentioned metaheuristic-based positioning methods are compared in terms of different aspects, like initial positioner, optimizer, strengths, limitations, and published year, in Table A1.

## 3. Preliminaries

This section of this paper is dedicated to introducing the algorithms used in the development of the proposed method, which includes Artificial Jellyfish Search (JS), Artificial Rabbits Optimization (ARO), and Distance Vector-Hop (DV-Hop). This section also describes the network model and fitness function used in the proposed algorithm.

### 3.1. JS

The JS is a recently developed metaheuristic algorithm developed based on the jellyfish lifestyle in nature. Four concepts are discussed in the JS: ocean current, passive motion, active movement, and time control. This subsection briefly defines the concepts and provides their mathematical formulations; more details of the JS parts are provided in [25]. In general, jellyfish live in swarms and exhibit three movements: inside swarm movement, active motion, and passive motion. In the JS, a jellyfish swarm is generated using the chaos theory to enhance the distribution of jellyfish in the search space. Equation (1) expresses the swarm generation (initialization phase) of the JS algorithm mathematically.

(1) $$X_{i}=LB+LV\times (UB-LB)$$

where $X_{i}$ is the position of ith jellyfish in the d-dimensional space, LB contains the lower boundaries, UB is the upper boundaries of the problem space, and LV is a vector with values obtained by the Logistic chaotic map. Equation (2) presents the Logistic chaotic map.

(2) LV=α.LV×(1−LV)

**Figure 1:**
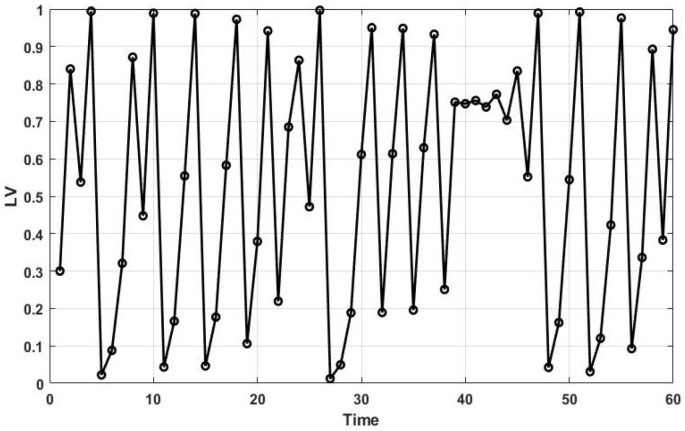
The distribution of the logistic chaotic map in 60-time slots.

It is noteworthy that the initial chaotic value (${LV}_{0}$) is between 0 and 1 and is not equal to 0, 0.25, 0.5, 0.75, or 1. Also, the α is a coefficient. Figure 1 represents the distribution of the chaotic values produced by the Logistic map with α=4 and ${LV}_{0}=0.3$ over 60 iterations.

Once the swarm is created and the position of the jellyfish is initialized, a parameter named time control (c) is calculated to determine how to update the position of the jellyfish. According to the c, a jellyfish decides to follow the ocean current or perform a motion within the swarm. Equation (3) presents the formula for the time control parameter.

(3) $$c=\left|\left(1-\frac{c_{i}}{m_{i}}\right)\times 2r_{1}-1\right|$$

where $c_{i}$ is the current iteration of the algorithm, $m_{i}$ is the maximum number of iterations, and $r_{1}$ is a random number in (0,1).

**Figure 2:**
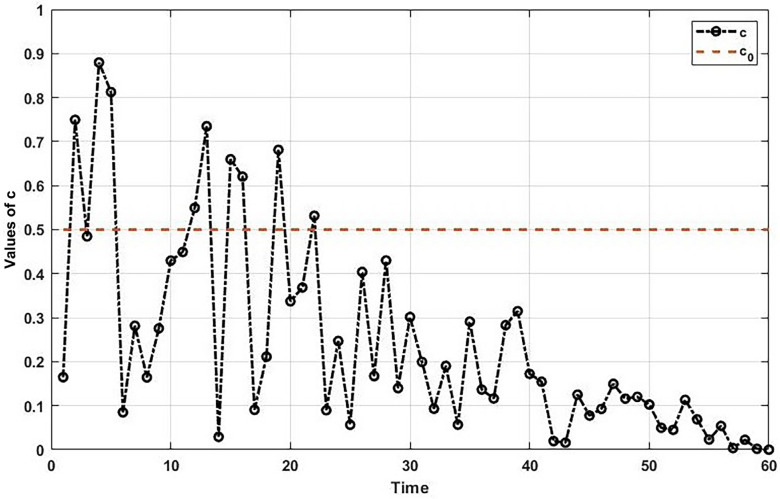
Values of the time control parameter (c) over 60 timeslots.

The ocean currents contain a significant amount of food sources that attract jellyfish. However, the amount of nutrition in the ocean current decreases over time, and the jellyfish is forced to migrate to another ocean current. The values of c over 60 iterations are demonstrated in Figure 2.

Along with the time control parameter, a decision parameter named $c_{0}$ is defined in the JS, which is considered 0.5. When $c\geq c_{0}$, the jellyfish will follow the ocean current and update its position using Equation (4).

(4) $$X_{i}=X_{i}+r_{2}\times Dir$$

In Equation (4), $X_{i}$ is the position of ith jellyfish, $r_{2}$ is a randomly-selected number within (0,1), and Dir is the direction of the ocean current computed as follows:

(5) $$Dir=\frac{1}{N}\sum_{i=1}^{N}{Dir}_{i}=\frac{1}{N}\sum \left(X_{B}-e_{c}{\cdot \;X}_{i}\right)=X_{B}-e_{c}\times \frac{\sum X_{i}}{N}=X_{B}-e_{c}\times \mu$$

(6) $$e_{c}=r_{3}\times \beta$$

where N is the jellyfish counts, $X_{B}$ is the position of the best jellyfish in the swarm, $e_{c}$ is the attraction govern factor, β=3 is the distribution coefficient, and μ is calculated by Equation (7).

(7) $$\mu =\frac{1}{N}\sum_{i=1}^{N}X_{i}$$

Equation (5) can be rewritten as below:

(8) $$Dir=X_{B}-df$$

(9) $$df=e_{c}\cdot \mu$$

A normal spatial distribution assumption states that jellyfish are scattered around the average position of jellyfish in the swarm. As a result, the df can be redefined as follows:

(10) $$df\;=\;\beta \times \;\sigma \;\times r_{4}$$

where $r_{4}$ is a random number in (0,1), and σ is the uniform distribution, which is obtained by Equation (11).

(11) $$\sigma =r_{4}\times \mu$$

Regarding Equations (6) and (9), the Dir can be simplified as below:

(12) $$Dir=X_{B}-r_{3}\times \beta \cdot \mu$$

When $c<c_{0}$, the jellyfish conduct two movements within the swarm: passive and active motions. Through the passive motion, which is expressed mathematically in Equation (13), the jellyfish moves around its current position.

(13) $$X_{i}=X_{i}+\gamma \times r_{5}\times (UB-LB)$$

In Equation (13), γ is a parameter that specifies the movement length. With the passage of time, jellyfish perform active motion. In this motion, the jellyfish updates its position regarding the position of another jellyfish in the swarm. Each time, a jellyfish selects a random jellyfish from the current swarm as its destination. Then, the jellyfish evaluates the food resources at the destination. If the food resources in the destination point are more than in the current position, the jellyfish moves towards the destination. Otherwise, the jellyfish moves against the destination position. Equation (14) expresses the active motion of JS mathematically.

(14) $$X_{i}=X_{i}+r_{5}\cdot step$$

(15) $$step=\left\{\begin{array}{c} X_{i}-X_{j}\;\;\;\;\;\;\;\;\;\;\;\;\;\;\;\;\;F_{j}\geq \;F_{i}\\ X_{j}-X_{i}\;\;\;\;\;\;\;\;\;\;\;\;\;\;\;\;\;\;F_{j}<F_{i} \end{array}\right.$$

where $X_{j}$ is the position of the target jellyfish and $F_{j}$ is the food amount of the destination point. The pseudocode of the JS algorithm is presented in Algorithm 1.
**Algorithm 1.** The pseudocode of JS Inputs: number of search agents (N), number of iterations ($m_{i}$), initial value of chaotic map (${LV}_{0}$), problem dimension (d), and lower (LB) and upper (UB) boundaries of search spaceOutputs: the best jellyfish ($X_{B}$) and its fitness ($F_{B}$)// Swarm generationi=1while (i≤N)   Initialize $X_{i}$ using Equation (1)   Calculate $F_{i}$   i=i+1end whileFind $X_{B}$ and $F_{B}$$c_{i}=1$while ($c_{i}\leq m_{i}$)  i=1  while (i≤N)      Compute c using Equation (3)      if ($c\geq c_{0}$)        // Ocean current        Update $X_{i}$ using Equation (4)      else        if (r≥1−c)        // Passive motion        Update $X_{i}$ using Equation (13)        else        // Active motion        Calculate step by Equation (15)        Update $X_{i}$ using Equation (14)        end if      end if      Update $F_{i}$      i=i+1  end for  Update $X_{B}$ and $F_{B}$  $c_{i}=c_{i}+1$end whileReturn $X_{B}$ and $F_{B}$

### 3.2. ARO

The ARO is a new born metaheuristic optimization algorithm developed based on the survival strategies of rabbits in nature. The ARO algorithm has adequate exploration and exploitation capabilities. Hence, despite its newness, it has been applied to various problems and has manifested promising results. This subsection introduces the different parts of the ARO and their mathematical formulations; more details about its motivation are presented in [26]. In the ARO, two kinds of behaviors are considered: detour foraging and random hiding. The rabbits conduct these strategies alternately regarding the energy parameter. The strategies and energy parameters of ARO are explained in the following.

The ARO algorithm, like other meta-heuristic algorithms, begins by establishing an initial population. In the initial population, rabbits are randomly scattered in the problem space using Equation (16).

(16) $$X_{i}=r_{6}\cdot \left[UB-LB\right]+LB$$

**Figure 3:**
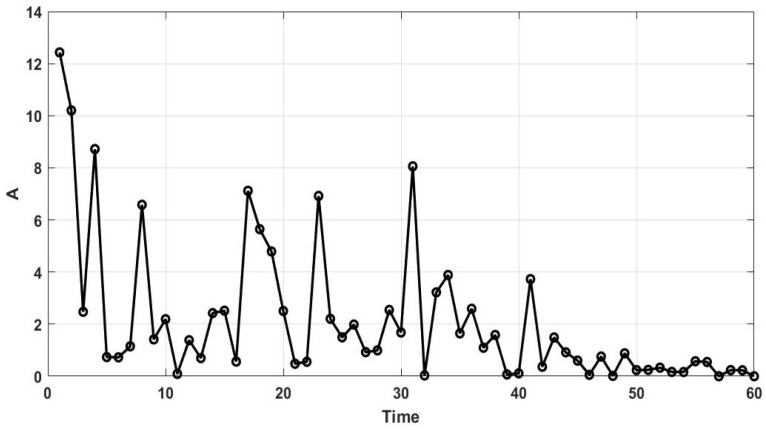
Values of A over 60 iterations.

Afterward, the energy shrink parameter (A) is calculated for each rabbit to predict their movement strategy. When the energy of rabbits is high, they perform the detour foraging strategy. However, by reducing the energy of rabbits in later iterations, they accomplish the random hiding strategy. Equation (17) simulates the energy shrink parameter of rabbits.

(17) $$A=4F\times \left[1-\frac{c_{i}}{m_{i}}\right]$$

(18) $$F=ln\left(\frac{1}{r_{7}}\right)$$

where $c_{i}$ is the current iteration, $m_{i}$ in the maximum number of iterations, and $r_{7}$ is a random number in the interval of (0,1). The values of the energy shrink parameter over 60 iterations are illustrated in Figure 3.

Considering the values of *A* depicted in Figure 3, it can be observed that the energy of rabbits is high in early iterations. Therefore, the rabbits are likely to do detour foraging. As time passes, the energy of rabbits diminishes, and they prefer random hiding. To imitate this process, it is stipulated in the ARO that, when the value of *A* is greater than one, the rabbit performs detour foraging and updates its position by Equation (19). Through detour foraging, the rabbits explore other rabbits’ territory and prevent their territory from being detected by predators.

(19) $$X_{i}=X_{j}+R\cdot (X_{i}-X_{j})+Z$$

(20) R=L·Q

(21) $$Z=n_{1}\times round\left(\frac{0.05+r_{8}}{2}\right)$$

(22) $$Q=\left\{\begin{array}{c} 1\;\;\;\;\;\;\;\;\;k==g\left(l\right)\;\;\\ 0\;\;\;\;\;\;\;\;otherwise\;\;\; \end{array}\right.$$

(23) $$L=sin\left(2\pi r_{9}\right)\times H$$

(24) $$H=\left[e-e^{{\left(\frac{c_{i}-1}{m_{i}}\right)}^{2}}\right]$$

(25) $$h_{1}=\left\lceil r_{10}\times d\right\rceil$$

(26) g=randperm(d)

where $X_{i}$ is the position of the current rabbit, $X_{j}$ is the position of a randomly-selected rabbit from the search space, $i,j\in \left\{1,2,\ldots ,N\right\},$ and i≠j. The $n_{1}$ is a random number generated with a normal distribution, k∈{1,2,…, d}, $l\in \{1,2,\ldots ,h_{1}\}$, d is the dimension of the search space, $\left\lceil \;.\;\right\rceil$ is the ceiling function, $r_{8}$, $r_{9}$, and $r_{10}$ are random numbers between (0,1). Also, the randperm function returns a random permutation.

Likewise, in the condition that *A* is less than or equal to one, the second approach, random hiding, is adopted. In nature, rabbits dig different holes in their nests to hide and avoid predators. Through random hiding, the rabbits select a random burrow to hide. Through this behavior, the position of a rabbit in the ARO is updated using Equation (27).

(27) $$X_{i}=X_{i}+R\times \left(r_{11}\times b_{r}-X_{i}\right)$$

In Equation (27), $r_{11}$ is a random number in (0,1) and $b_{r}$ is the randomly chosen borrow around the nest. In the ARO, a burrow is obtained as follows:

(28) $$b_{i}=\left[n_{2}\cdot H\right]\times [g_{r}\cdot X_{i}]+\;X_{i}$$

(29) $$H=\frac{m_{i}-c_{i}-1}{m_{i}}$$

(30) $$g_{r}=\left\{\begin{array}{c} 1\;\;\;\;\;\;\;\;\;\;k==h_{2\;\;\;\;}\;\\ 0\;\;\;\;\;\;\;\;otherwise\;\; \end{array}\right.$$

(31) $$h_{2}=\left\lceil r_{12}\times d\right\rceil$$

where the $b_{i}$ is a burrow dug by the ith rabbit, $r_{12}$ is a random within (0,1), $k\in \left[1,2,\ldots ,\;d\right]$, and $n_{2}$ is a random number acquired by the normal distribution. Algorithm 2 represents the pseudocode of the ARO algorithm.

### 3.3. DV-Hop

The DV-Hop is a well-known range-free positioning method, which is used in much research and real-life applications. The DV-Hop is one of the most popular positioning methods due to the simplicity of its implementation, proper execution time, no need for additional equipment, and less energy consumption [27]. This positioning algorithm estimates the position of unknown wireless objects regarding anchors (devices with prior position information) [28,29]. It is worth noting that the equations presented in the rest of this subsection are an adapted version of the basic DV-Hop for 3D environments.
**Algorithm 2.** The pseudocode of AROInputs: number of search agents (N), number of iterations ($m_{i}$), problem dimension (d), and lower (LB) and upper (UB) boundaries of search spaceOutputs: the best rabbit ($X_{B}$) and its fitness ($F_{B}$)//Initializationi=1while (i≤N)   Initialize $X_{i}$ by Equation (16)   Calculate $F_{i}$   i=i+1end forFind $X_{B}$ and $F_{B}$$c_{i}=\;1$while ($c_{i}<m_{i}$)   i=1   while (i≤N)      Calculate energy shrink (A) by Equation (17)      if A>1        //Detour foraging        Update $X_{i}$ using Equation (19)      else        //Random hiding        Update $X_{i}$ using Equation (27)      end if      Compute $F_{i}$      i=i+1   end for   Update $X_{B}$ and $F_{B}$   $c_{i}=\;c_{i}+\;1$end whileReturn $X_{B}$ and $F_{B}$

**Figure 4:**
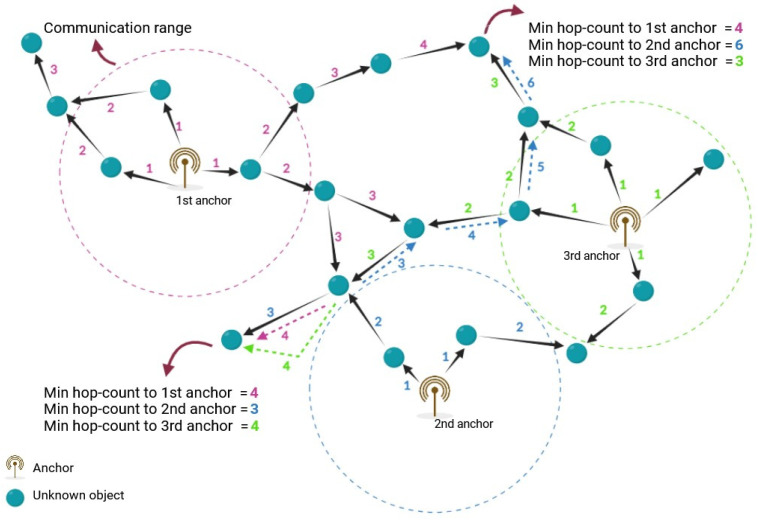
Determining the hop counts in the DV-Hop.

The first phase of the DV-Hop algorithm is determining the minimum number of hops between unknown devices and anchors. In this regard, each anchor broadcasts a packet containing its position information to its neighbors. This packet also includes a hop-counter field with an initial value of one. Unknown devices in the anchor’s coverage area receive this packet. After storing the anchor’s location information, these devices add one unit to the value of the hop-counter field and rebroadcast the new packet to the nearby devices. Unknown devices that have received more than one packet from one or more anchors keep only the packet with the lowest hop count and drop the remaining packets. At the end of the initial phase, all unknown devices recognize their minimum hop counts from the anchors. This process is demonstrated in Figure 4.

Once the minimum hop counts from the unknown devices to the anchors are acquired, the average hop count is calculated using Equation (32) in the second phase.

(32) $${AHC}_{i}=\frac{\sum_{j=1}^{N_{A}}\sqrt{{\left(X_{A_{i}}-X_{A_{j}}\right)}^{2}+{\left(Y_{A_{i}}-Y_{A_{j}}\right)}^{2}+{\left(Z_{A_{i}}-Z_{A_{j}}\right)}^{2}}}{\sum_{j=1}^{N_{A}}{MHC}_{i,j}}$$

(33) $${Dist}_{i,j}={AHC}_{i}\times {MHC}_{i,j}$$

(34) $$\left\{\begin{array}{cccccc} {\left({X_{A_{1}}-X}_{ui}\right)}^{2} & + & {\left(Y_{A_{1}}-Y_{ui}\right)}^{2} & + & {\left(Z_{A_{1}}-Z_{ui}\right)}^{2} & ={{(Dist}_{A_{1},u_{i}})}^{2}\\ {\left({X_{A_{2}}-X}_{ui}\right)}^{2} & + & {\left({Y_{A_{2}}-Y}_{ui}\right)}^{2} & + & {\left(Z_{A_{2}}-Z_{ui}\right)}^{2} & ={{(Dist}_{A_{2},u_{i}})}^{2}\\ \vdots & & \vdots & & \vdots & \vdots \\ {\left({X_{A_{k}}-X}_{ui}\right)}^{2} & + & {\left({Y_{A_{k}}-Y}_{ui}\right)}^{2} & + & {\left(Z_{A_{k}}-Z_{ui}\right)}^{2} & ={{(Dist}_{A_{k},u_{i}})}^{2} \end{array}\right.$$

(35) $$\left\{\begin{array}{c} \;\;\;X_{A_{1}}^{2}-X_{A_{j}}^{2}-2\left(X_{A_{1}}-X_{A_{k}}\right)X_{u_{i}}+Y_{A_{1}}^{2}-Y_{A_{k}}^{2}-2(Y_{A_{1}}-Y_{A_{k}})Y_{u_{i}}+Z_{A_{1}-}^{2}Z_{A_{k}}^{2}-2(Z_{A_{1}}-Z_{A_{k}})Z_{u_{i}}={\left({Dist}_{A_{1},u_{i}}\right)}^{2}-{\left({Dist}_{A_{k},u_{i}}\right)}^{2}\\ \;\;\;X_{A_{2}}^{2}-X_{A_{k}}^{2}-2\left(X_{A_{2}}-X_{A_{k}}\right)X_{u_{i}}+Y_{A_{2}}^{2}-Y_{A_{k}}^{2}-2(Y_{A_{2}}-Y_{A_{k}})Y_{u_{i}}+Z_{A_{1}-}^{2}Z_{A_{k}}^{2}-2(Z_{A_{2}}-Z_{A_{k}})Z_{u_{i}}={\left({Dist}_{A_{2},u_{i}}\right)}^{2}-{\left({Dist}_{A_{k},u_{i}}\right)}^{2}\\ \;\;\;\;\vdots \;\vdots \;\vdots \;\vdots \;\vdots \;\vdots \;\vdots \;\vdots \;\vdots \;\vdots \;\vdots \\ X_{A_{k-1}}^{2}-X_{A_{k}}^{2}-2\left(X_{A_{k-1}}-X_{A_{k}}\right)X_{u_{i}}+Y_{A_{k-1}}^{2}-Y_{A_{k}}^{2}-2\left(Y_{A_{k-1}}-Y_{A_{k}}\right)Y_{u_{i}}+Z_{A_{k-1}}^{2}-Z_{A_{k}}^{2}-2(Z_{A_{k-1}}-Z_{A_{k}})Z_{u_{i}}={\left({Dist}_{A_{k-1},u_{i}}\right)}^{2}-{\left({Dist}_{A_{k},u_{i}}\right)}^{2} \end{array}\right.$$

(36) A·P=B

(37) $$P={\left(A^{T}\cdot A\right)}^{-1}\times A^{T}\cdot B$$

(38) $$A=\left[\begin{array}{ccc} 2\left(X_{A_{1}}-X_{A_{k}}\right) & 2(Y_{A_{1}}-Y_{A_{k}}) & 2(Z_{A_{1}}-Z_{A_{k}})\\ 2\left(X_{A_{2}}-X_{A_{k}}\right) & 2\left(Y_{A_{2}}-Y_{A_{k}}\right) & 2(Z_{A_{2}}-Z_{A_{k}})\\ \vdots & \;\;\;\;\;\vdots & \vdots \\ 2(X_{A_{k-1}}-X_{A_{k}}) & 2(Y_{A_{k-1}}-Y_{A_{k}}) & 2(Z_{A_{k-1}}-Z_{A_{k}}) \end{array}\right]$$

(39) $$P=\left[\begin{array}{c} X_{A_{1}}^{2}-X_{A_{k}}^{2}+Y_{A_{1}}^{2}-Y_{A_{k}}^{2}+Z_{A_{1}-}^{2}Z_{A_{k}}^{2}-{\left({Dist}_{A_{1},u_{i}}\right)}^{2}+{\left({Dist}_{A_{k},u_{i}}\right)}^{2}\\ X_{A_{2}}^{2}-X_{A_{k}}^{2}+Y_{A_{2}}^{2}-Y_{A_{k}}^{2}+Z_{A_{1}-}^{2}Z_{A_{k}}^{2}-{\left({Dist}_{A_{2},u_{i}}\right)}^{2}+{\left({Dist}_{A_{k},u_{i}}\right)}^{2}\\ \;\vdots \;\vdots \;\vdots \;\vdots \;\vdots \;\vdots \;\vdots \;\vdots \\ X_{A_{k-1}}^{2}-X_{A_{k}}^{2}+Y_{A_{k-1}}^{2}-Y_{A_{k}}^{2}+Z_{A_{k-1}}^{2}-Z_{A_{k}}^{2}-{\left({Dist}_{A_{k-1},u_{i}}\right)}^{2}+{\left({Dist}_{A_{k},u_{i}}\right)}^{2} \end{array}\right]$$

In Equation (32), ${AHC}_{i}$ is the average hop count of ith anchor, $\left[X_{A_{i}},Y_{A_{i}},Z_{A_{i}}\right]$ is the coordinate of ith anchor, ${MHC}_{i,j}$ is the minimum hop count between ith and jth anchors, and $N_{A}$ is the number of deployed anchors. In the following phase, the distance of unknown objects from the anchors is computed by Equation (33), where ${Dist}_{i,j}$ is the distance of ith unknown object from the jth anchor and ${MHC}_{i,j}$ is the minimum number of hops between the ith unknown object and jth anchor.

Eventually, the position of unknown objects is estimated using the multilateration method in the fourth phase. In the Multilateration, k equations are formed as follows for each unknown wireless object. In Equation (34), $\left[X_{A_{k}},Y_{A_{k}},Z_{A_{k}}\right]$ is the coordinates of the kth anchor, $\left[X_{ui},Y_{ui},Z_{ui}\right]$ is the coordinates of the ith unknown object, and ${Dist}_{A_{k},u_{i}}$ is the distance between the kth anchor and ith unknown object. Equations formed in Equation (34) can be expanded as Equation (35). The expanded equations can also be expressed by matrix rules as Equations (36)–(40), where P is the estimated position of ith unknown object.

### 3.4. Network Model and Problem Definition

This section introduces the 3D IoT network utilized in the current paper and defines the positioning problem as an optimization problem in such networks. Like most energy-based monitoring IoT networks, wireless objects in the network adopted in the present paper are unaware of their geographic location because they are deployed randomly. These wireless objects are called unknown objects and are denoted as below:

(40) $$UO=\left[\begin{array}{cc} x_{1} & \begin{array}{cc} y_{1} & \;\;\;\;\;z_{1} \end{array}\\ \begin{array}{c} \begin{array}{c} x_{2}\\ \vdots \end{array}\\ x_{N_{UO}} \end{array} & \begin{array}{cc} \begin{array}{c} \begin{array}{c} y_{2}\\ \vdots \end{array}\\ y_{N_{UO}} \end{array} & \begin{array}{c} \begin{array}{c} z_{2}\\ \vdots \end{array}\\ z_{N_{UO}} \end{array} \end{array} \end{array}\right]$$

where the $\left[x_{i},y_{i},z_{i}\right]$ is the position of ith unknown object and $N_{UO}$ is the number of unknown objects in the network. Unknown objects in the network can be of various types, including surveillance cameras, thermal sensors, motion sensors, humidity-measuring devices, fire detectors, etc.

Moreover, a small number of devices are deployed in the network, and their locations are specified using GPS or manual configuration. These devices are named anchors and are stated as follows:

(41) $$AD=\left[\begin{array}{cc} x_{1} & \begin{array}{cc} y_{1}\;\;\;\;\; & z_{1} \end{array}\\ \begin{array}{c} \begin{array}{c} x_{2}\\ \vdots \end{array}\\ x_{N_{A}} \end{array} & \begin{array}{cc} \begin{array}{c} \begin{array}{c} y_{2}\\ \vdots \end{array}\\ y_{N_{A}} \end{array} & \begin{array}{c} \begin{array}{c} z_{2}\\ \vdots \end{array}\\ z_{N_{A}} \end{array} \end{array} \end{array}\right]$$

where $N_{A}$ is the number of anchors in the network. Additionally, this paper assumes that both unknown objects and anchors in the network model are equipped with devices capable of sending and receiving data within a limited communication range.

The proposed algorithm uses the 3D-DV-Hop model to estimate the UO by the AD and Equation (37). However, the estimated positions by the 3D-DV-Hop have a significant error due to the imbalance of hop lengths in 3D networks. For precise estimations in 3D IoT networks, an enhanced hybrid meta-heuristic algorithm based on the JS and ARO algorithms is integrated with 3D-DV-HOP to diminish the Positioning Mean Square Error (PMSE). The PMSE of estimated positions is calculated using Equation (42).

(42) $$minimize:\;PMSE=\frac{\sum_{1}^{N_{UO}}\sqrt{{\left(X_{i_{est}}-X_{i_{act}}\right)}^{2}+{\left(Y_{i_{est}}-Y_{i_{act}}\right)}^{2}+{\left(Z_{i_{est}}{-Z}_{i_{act}}\right)}^{2}}}{N_{UO}}$$

where [$X_{i_{est}},\;Y_{i_{est}},\;Z_{i_{est}}]$ is the obtained coordinates of ith unknown object and $\left[X_{i_{act}},Y_{i_{act}},Z_{i_{act}}\right]$ is the actual position of ith unknown object. The proposed hybrid meta-heuristic, described in the following section, reduces the PMSE by modifying initial positions estimated by the 3D DV-Hop model.

## 4. Proposed Algorithm

**Figure 5:**
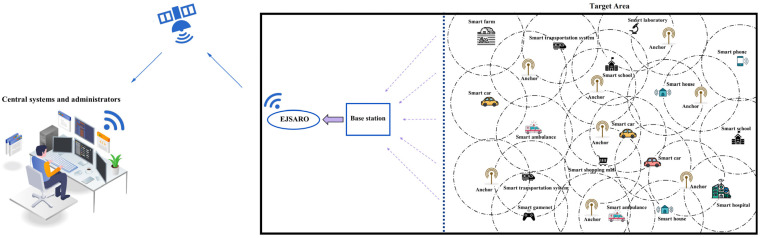
An overview of the proposed positioning framework.

In this paper, the DV-Hop algorithm is extended to be applicable to 3D environments. However, distance estimations between unknown IoT objects and anchors are still prone to error, as the step size is approximately determined by the distance between anchors. Also, the environmental noises and their destructive effect on the transmitted packets cause the estimated locations to contain a significant error, especially in large-scale 3D IoT networks. In this regard, several methods have been proposed to improve positioning accuracy, among which methods based on meta-heuristic algorithms demonstrated favorable results. Therefore, in this research, by taking advantage of JS and ARO algorithms, an enhanced hybrid meta-heuristic algorithm called EJSARO has been introduced and integrated with 3D DV-Hop. This section introduces the different parts of the proposed EJSARO algorithm and expresses its implementation details. A high-level representation of the proposed positioning framework is illustrated in Figure 5.

In the beginning, the desired target environment is investigated, and the number and type of IoT devices and anchors are specified and distributed randomly in the desired area. Next, the exact location of the anchors is determined by GPS, and the distance between them is calculated. Afterward, the number of hops between the unknown nodes and the anchors is obtained by the identification packets broadcast by the anchors. Once the hop counts between the anchors are acquired, the average hop size is computed by dividing the distance between the anchors by the hop counts between them. Then, the distance of each unknown device to the anchors is calculated by multiplying the number of hops to the anchor by the average step size. Eventually, the initial position of the unknown nodes is estimated by forming and solving Equation (34). In the following, the EJSARO rectifies the initially estimated positions and improves the location information.

In the EJSARO, the JS and ARO are hybridized, and several improvements are applied to improve searchability and local departure capability. Also, it has been made to avoid the significant increase in complexity and execution time while improving the capabilities of EJSARO. For instance, the time control parameter (Equation (3)), ocean current (Equation (4)), and passive motion (Equation (13)) are omitted from the original JS to lighten EJSARO, and the active motion (Equation (14)) is redefined as below:

(43) $$X_{i}\;=\;X_{B}\;+\;r_{5}\cdot step$$

Likewise, the energy shrink parameter (Equation (17)) and random hiding strategy (Equation (27)) are removed in the EJSARO, and the Detour foraging strategy (Equation (19)) is modified as follows:

(44) $$X_{i}=X_{B}+R\cdot (X_{i}-X_{j})+Z$$

Equations (43) and (44) enhance the exploitation capability of the EJSARO. In this case, the solutions are prone to becoming trapped in local optima. As a result, a local departure mechanism has been provided in EJSARO to detect and depart the solutions trapped in local optimums. In the local departure mechanism, a counter is defined for each solution. Every time the solution is updated, if the new position of the solution is better than its current position, the position of the solution is updated, and its counter is reset. Otherwise, the movement is not taken into account, and the previous position of the solution is preserved, and the counter of that solution is increased by one unit. Once the counter of a solution exceeds a predefined threshold, the position of the solution is initialized by the 3D DV-Hop algorithm.

The threshold value has a significant effect on the efficiency of the local departure mechanism and can adjust the exploration and exploitation capabilities. However, if small values are considered as the threshold, the exploration increases abnormally, and the convergence of the algorithm is weakened. Also, if large values are assigned to it, the exploitation and convergence of the algorithm are increased, but the solutions remain in the local optima for a while, or in the worst case, cannot leave the local optima. Consequently, the threshold should be adjusted carefully. After the solutions are updated, their boundaries are checked and rectified using Equation (45).

(45) $$X_{i}=\varphi \cdot \left[r_{13}\times (UB-LB)+LB\right]+\left[X_{i}\times \bar{\varphi }\right]$$

(46) $$\varphi =\left(X_{i}>UB\right)+(X_{i}<LB)$$

In the following, a new phase has been introduced to EJSARO to strengthen its search capabilities as much as possible. Through this phase, the position of solutions is updated using the Levy flight function as below:

(47) $$X_{i}=X_{i}+{Lv}_{i}$$

(48) $${Lv}_{i}=\alpha \times \frac{\beta }{{\left|\gamma \right|}^{1/\eta }}$$

(49) $$\beta =N(0,\sigma_{\beta }^{2})$$

(50) $$\gamma =N(0,\sigma_{\gamma }^{2})$$

where ${Lv}_{i}$ is the value produced by the Levy flight, $N(0,\sigma_{\beta }^{2})$ is the normal distribution function with a standard deviation of $\sigma_{\beta }$ and $\sigma_{\gamma }$, and α=0.05 is a coefficient. Also, the $\sigma_{\gamma }$ is equals to 1, and the $\sigma_{\beta }$ is obtained using Equation (51).

(51) $$\sigma_{\beta }={\left(\frac{\Gamma (1+\vartheta )\times sin(\frac{\vartheta \cdot \pi }{2})}{\vartheta \cdot \Gamma (\frac{1+\vartheta \;}{2})\times {2\;}^{\frac{\;\vartheta -1\;}{2}}}\right)}^{\frac{1\;}{\vartheta }}$$

where ϑ=1.5. The local departure mechanism is also recalled in this phase after updating the solutions by the Levy flight.

As the positioning of unknown IoT objects intentionally requires exploitation, the exploitation ability has been augmented more than exploration in EJSARO. In this situation, the best solution found so far in the population has a notable impact on population guiding and the final result achieved by the algorithm. Accordingly, a local search procedure is provided in EJSARO to improve the best solution. In the local search procedure, the best solution found so far in the population is updated by Equation (52), and the greedy selection process is adopted.

(52) $$X_{B}=X_{B}+\delta \cdot \left[X_{j}-X_{k}\right]$$

(53) $$\delta =\left[r_{13}-0.5\right]\times \left[1-\frac{c_{i}}{m_{i}}\right]$$

where $r_{13}$ is a random number in (0,1), $c_{i}$ is the current iteration, $m_{i}$ in the maximum number of iterations, and $X_{j}$ and $X_{k}$ are randomly selected solutions from the current population. Algorithm 3 exposes the pseudocode of the proposed local search procedure in the EJSARO.
**Algorithm 3.** The pseudocode of the proposed local search procedureInputs: search agents (X), the best search agent ($X_{B}$), current iteration ($c_{i}$), maximum number of iterations ($m_{i}$), and lower (LB) and upper (UB) boundaries of search spaceOutputs: the best search agent ($X_{B}$) and its fitness ($F_{B}$)for (i=1 to φ)   Select $X_{j}$ and $X_{k}$ randomly from the X   Calculate δ using Equation (53)   Obtain ${newX}_{B}$ using Equation (52)   Check and rectify the ${newX}_{B}$ using Equation (45)   Compute ${newF}_{B}$ by Equation (42)   if ${newF}_{B}<F_{B}$      $X_{B}=\;{newX}_{B}$      $F_{B}={newF}_{B}$   end ifend forReturn $X_{B}$ and $F_{B}$

The φ is a parameter that controls the local search cycle. The larger the value of φ is, the better the final solution of the algorithm will be, but the execution time will increase relatively. The value of φ is considered 5 in the EJSARO. It is also worth mentioning that, in EJSARO, solutions are initialized by Equation (40) instead of Equations (1) and (16). Algorithm 4 presents the pseudocode of the proposed EJSARO-based positioning framework.
**Algorithm 4.** The pseudocode of the EJSAROInputs: number of search agents (N), number of iterations ($m_{i}$), problem dimension (d), and lower (LB) and upper (UB) boundaries of search spaceOutputs: the best search agent ($X_{B}$) and its fitness ($F_{B}$)//Initializationi=1;    $c_{i}=\;1$;while (i≤N)   Initialize $X_{i}$ by Equation (39)   Calculate $F_{i}$ using PMSE formulated by Equation (42)   ${ct}_{i}=0$;    i=i+1;end forFind $X_{B}$ and $F_{B}$//Optimizationwhile ($c_{i}\leq m_{i}$)   i=1   while (i≤N)     j=randint(N);     if $rand\left(0,1\right)<0.5$      //Modified Detour foraging (ARO)      Calculate R, Z, and L using Equations (20), (21), and (23), respectively      ${newX}_{i}=X_{B}+R.(X_{i}-X_{j})+Z$     else      //Modified Random hiding (JS)      Compute step using Equation (15)      ${newX}_{i}\;=\;X_{B}\;+\;r_{5}.step$     end if     //Check and rectify the new solution     Calculate φ using Equation (46)     $newX_{i}$ = $\varphi .\left[r_{13}\;\times (UB-LB)+LB\right]\;\times \;+\;\left[{newX}_{i}\;\times \bar{\varphi }\right]$     //Elitism (greedy selection)     Compute ${newF}_{i}$ using Equation (42)     if $\left({newF}_{i}<\;F_{i}\right)$      $X_{i}=\;{newX}_{i}$;    $F_{i}={newF}_{i}$;    ${ct}_{i}=0$;     else      ${ct}_{i}=\;{ct}_{i}+1$     end     if $\left({newF}_{i}<\;F_{B}\right)$      $X_{B}=\;{newX}_{i}$;    $F_{B}={newF}_{i}$;     end     //Levy flight-based motion     Calculate ${Lv}_{i}$ using Equation (48)     ${newX}_{i}=X_{i}+{Lv}_{i}$     //Check and rectify the new solution     $newX_{i}$ = $\varphi .\left[r_{13}\;\times (UB-LB)+LB\right]\;\times \;+\;\left[{newX}_{i}\;\times \bar{\varphi }\right]$     //Elitism (greedy selection)     Compute ${newF}_{i}$ using Equation (43)     if $\left({newF}_{i}<\;F_{i}\right)$      $X_{i}=\;{newX}_{i}$;    $F_{i}={newF}_{i}$;    ${ct}_{i}=0$;     else      ${ct}_{i}=\;{ct}_{i}+1$     end     if $\left({newF}_{i}<\;F_{B}\right)$      $X_{B}=\;{newX}_{i}$;    $F_{B}={newF}_{i}$;     end     //Local departure mechanism     if $\left({ct}_{i}\geq Th\right)$      Reset$\;{ct}_{i}$ and reinitialize $X_{i}$ using Equation (39)     end     i=i+1   end for   //Proposed local search procedure   Improve $X_{B}$ using Equation (52) and the proposed local search   $c_{i}=\;c_{i}+\;1$end whileReturn $X_{B}$ and $F_{B}$

## 5. Simulation Results

To substantiate the strengths of the proposed 3D positioning framework and the effectiveness of the contributions, numerous simulations are conducted on twenty-four 3D IoT networks with varying specifications. The results are presented and discussed in the following subsections. It is noteworthy that all experiments are performed on the same system with the specifications provided in Table 1.

Considering that the metaheuristic-based methods are random in nature, each positioning method was applied twenty-five times independently to the networks, and the obtained results were recorded. Then, the set of algorithm results is compared numerically and graphically by multiple criteria.

### 5.1. Competitors

To affirm the advantages of the proposed positioning method over the existing methods, the results obtained by the EJSARO are compared with nine similar methods. In this regard, the 3D DV-Hop algorithm is integrated with the following algorithms, which are among the most reliable, accurate, and widely used metaheuristic algorithms, and are considered competitors of the EJSARO: Artificial Ecosystem-based Optimization [30] (AEO-based DV-Hop), Aquila Optimizer [31] (AO-based DV-Hop), Artificial Rabbits Optimization [26] (ARO-based DV-Hop), Dandelion Optimizer [32] (DO-based DV-Hop), Fox Optimizer [33] (FOX-based DV-Hop), Jellyfish Search optimizer [25] (JS-based DV-Hop), Sine Cosine Algorithm [34] (SCA-based DV-Hop), Tunicate Swarm Algorithm [35] (TSA-based DV-Hop), and Whale Optimization Algorithm [36] (WOA-based DV-Hop). The parameters of these algorithms, along with their values, are listed in Table 2, which are the suggested values by their developers.

### 5.2. Evaluation Metrics

In the comparisons, the results of the EJSARO have been compared with the competitors through multiple criteria, which are defined in this subsection. For each criterion, the results of the algorithms are initially compared numerically using the statistical criteria (the best and worst achieved solutions and the average, median, and standard deviation of the set of obtained results). Next, the results are visually plotted by the box plot diagrams. In the following, the impact of the investigated characteristic of the network is evaluated through bar and line graphs. Additionally, the convergence rate of the algorithms is visually compared.

The first evaluation criterion is the Mean Squared Error (MSE) (fitness function) and is formulated by Equation (54), where $N_{UO}$ is the number of unknown IoT devices, and ${[X}_{i_{act}},Y_{i_{act}},Z_{i_{act}}]$ and ${[X}_{i_{est}},Y_{i_{est}},Z_{i_{est}}]$ are the actual and estimated positions of ith unknown IoT device, respectively.

The second criterion is the Node Localization Error (NLE), which measures the positioning error of every single unknown IoT object. Equation (55) expresses the mathematical formulation of the NLE.

The third evaluation criterion is Average Localization Error (ALE) presented mathematically in Equation (56), where R is the communication range. Likewise, the Localization Error Variance (LEV) is considered the last evaluation criterion formulated mathematically by Equation (57).

(54) $$MSE=\frac{1}{N_{UO}}\sum_{1}^{N_{UO}}\sqrt{{\left(X_{i_{est}}-X_{i_{act}}\right)}^{2}+{\left(Y_{i_{est}}-Y_{i_{act}}\right)}^{2}+{\left(Z_{i_{est}}{-Z}_{i_{act}}\right)}^{2}}$$

(55) $${NLE}_{i}=\sqrt{{\left(X_{i_{est}}-X_{i_{act}}\right)}^{2}+{\left(Y_{i_{est}}-Y_{i_{act}}\right)}^{2}+{\left(Z_{i_{est}}{-Z}_{i_{act}}\right)}^{2}}$$

(56) $$ALE=\frac{\sum_{i=1}^{N_{UO}}\sqrt{{\left(X_{i_{est}}-X_{i_{act}}\right)}^{2}+{\left(Y_{i_{est}}-Y_{i_{act}}\right)}^{2}+{\left(Z_{i_{est}}{-Z}_{i_{act}}\right)}^{2}}}{N_{UO}\times R}$$

(57) $$LEV=\sqrt{\frac{\sum_{i=1}^{N_{UO}}{\left(\sqrt{{\left(X_{i_{est}}-X_{i_{act}}\right)}^{2}+{\left(Y_{i_{est}}-Y_{i_{act}}\right)}^{2}+{\left(Z_{i_{est}}{-Z}_{i_{act}}\right)}^{2}-ALE\times R}\right)}^{2}}{N_{UO}\times R^{2}}}$$

### 5.3. Networks

In this paper, to rigorously evaluate the performance of the proposed framework, sixteen 3D IoT network environments were carefully designed. These networks were constructed to represent a broad spectrum of deployment scenarios to test different aspects of the proposed framework, including scalability, robustness, and localization accuracy. The design parameters—such as network area, node density, anchor count, and communication range—were varied systematically across networks. Each network configuration was developed to reflect a distinct real-world deployment scenario, ranging from compact indoor-like setups to large-scale outdoor IoT systems.

In each scenario, sensor nodes were randomly distributed within the 3D environment using a uniform random generator, ensuring diverse topological structures and avoiding any bias in position estimation. Anchor nodes were also randomly placed but kept fixed across repeated runs of the same scenario to maintain comparability of results. This randomization strategy allows the algorithm to be evaluated over multiple heterogeneous topologies, reflecting real-world IoT deployments where node positions are rarely uniform or deterministic. Table 3 presents the specifications of the IoT networks.

Consequently, the experiments are organized in four scenarios; in each scenario, only one aspect of the network is variable, and the other characteristics are fixed. The following subsections provide the simulation results by scenario.

### 5.4. Networks with Different Deployment Area Size

**Figure 6:**
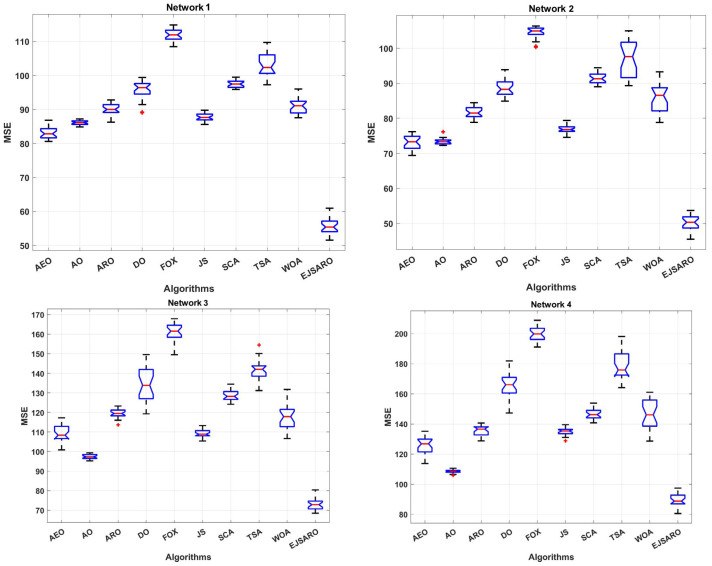
The box plot diagrams of the MSE results on Networks 1 to 4 (the red points are the outliers).

**Figure 7:**
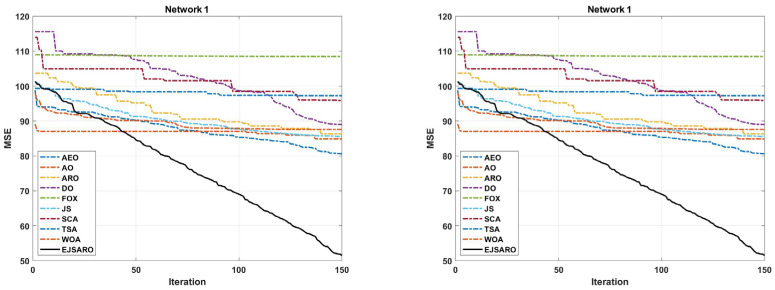

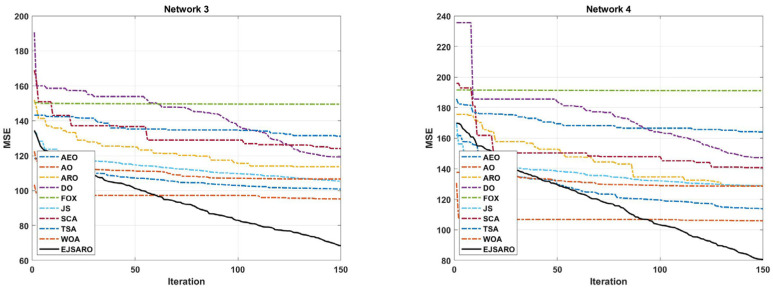
The best convergence of the algorithms in Networks 1 to 4.

This subsection presents the first set of experiments performed to evaluate the performance of the positioning algorithms. In these experiments, four three-dimensional IoT networks (Network 1 to Network 4) have been designed, and algorithms are applied to them to localize unknown objects. Networks 1 to 4 contain 200 IoT devices with unknown positions and 50 beacons with known locations. Also, the communication range (R) in these networks is set to 70 m. However, the dimensions of these networks are considered 350×350×150, 400×400×150, 450×450×150, and 500×500×150 cubic meters, respectively. Table 4 provides the statistical results of the algorithms in terms of MSE (fitness value). Additionally, Figure 6 and Figure 7 illustrate the box plots of algorithm results and the best convergence of algorithms in Networks 1 to 4, respectively.

According to Table 4 and Figure 6, which provide the statistical and box plot diagrams of results in terms of the MSE, the EJSARO outperformed the AEO, AO, ARO, DO, FOX, JS, SCA, TSA, and WOA algorithms in Networks 1 to 4. In these networks, the best and worst predictions of the EJSARO and the average and median of the obtained solutions are better than the compared algorithms. The box plots demonstrate that the set of solutions obtained by the EJSARO is much better than the other metaheuristic-based positioning models.

**Figure 8:**
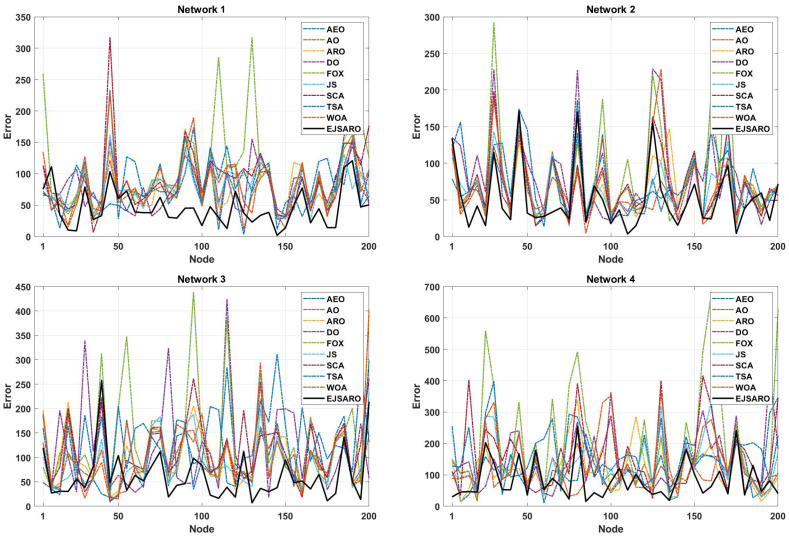
The NLE of algorithms in Networks 1 to 4.

It is noteworthy that the standard deviation of the EJSARO is more than competitors; nonetheless, a more accurate examination of box plots indicates that the EJSARO fluctuates in fewer MSE values. Additionally, the convergence diagrams of the algorithms, depicted in Figure 7, assert that the EJSARO reached lower MSE values in less time in Networks 1 to 4. Also, it can be concluded that the proposed model is able to obtain a continuous reduction pattern, which indicates its good performance in avoiding the local optima. Such a reduction requires high adequacy in both exploration and exploitation capabilities; in the case of a weakness in each of these two phases, the algorithm easily becomes stuck in local optimality. To provide a deeper examination, the best NLE values obtained by the algorithms are plotted in Figure 8.

**Figure 9:**
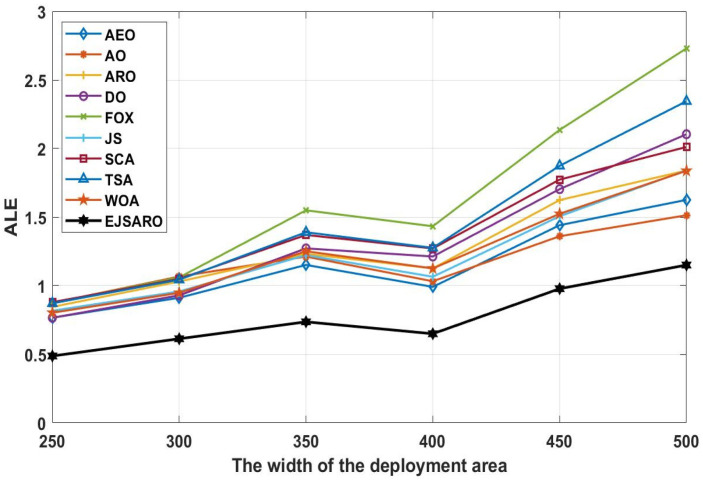
The best ALE values obtained by the algorithms on Networks with different scales.

According to the NLE graphs illustrated in Figure 8, it can be observed that the proposed model has achieved a lower error rate in most of the unknown objects. Also, the NLE peaks of the EJSARO’s estimations are much lower than similar algorithms in Networks 1 to 4, which reveals the higher reliability of the EJSARO and its excellent capability in discovering the position of network objects. In the following, the best ALE values of the algorithms on Networks 1 to 4 are compared in Figure 9.

**Figure 10:**
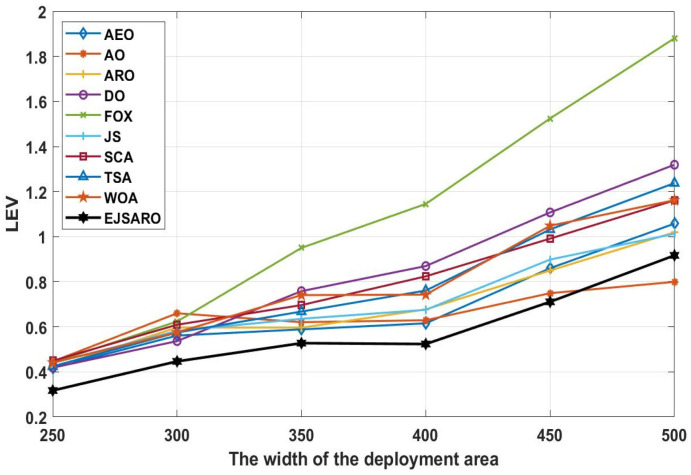
The best LEV values obtained by the algorithms on Networks with different scales.

Regarding the line graphs depicted in Figure 9, it can be noticed that the proposed positioning model has achieved much better results than other compared algorithms. This good performance has remained constant even with the increase in the size of the environment. Therefore, it can be claimed that the EJSARO has been able to obtain better results than similar models in environments of different sizes. Also, in these experiments, the FOX-based localization model had the worst performance among the algorithms. Moreover, it is obvious that the ALE and positioning errors grow with an increment in the size of the environment. The best LEV graphs are illustrated in Figure 10.

Considering the line graphs of Figure 10, it can be witnessed that the EJSARO positioning method performed well and achieved promising results. Thus, it can be asserted that the EJSARO outperformed its competitors regarding the LEV metric. However, by increasing the width of the environment to 500, the AO algorithm has been able to make a great leap and achieve better results than the compared algorithms. The most important thing is that, although the proposed model has not been able to have the best performance in a network of 500×500×150, it has still been able to maintain its search capabilities, which shows that the proposed algorithm has good search capabilities. Likewise, the LEV values represented in Figure 10 affirm the ALE and MSE results and indicate that the positioning error is raised by expanding the size of the target area. Regarding the numerical and visual MSE, NLE, ALE, and LEV results, it can be claimed that the EJSARO algorithm can estimate the position of IoT objects better than similar models, including AEO, AO, ARO, DO, FOX, JS, SCA, TSA, and WOA algorithms.

### 5.5. Networks with Different No. of IoT Devices

**Figure 11:**
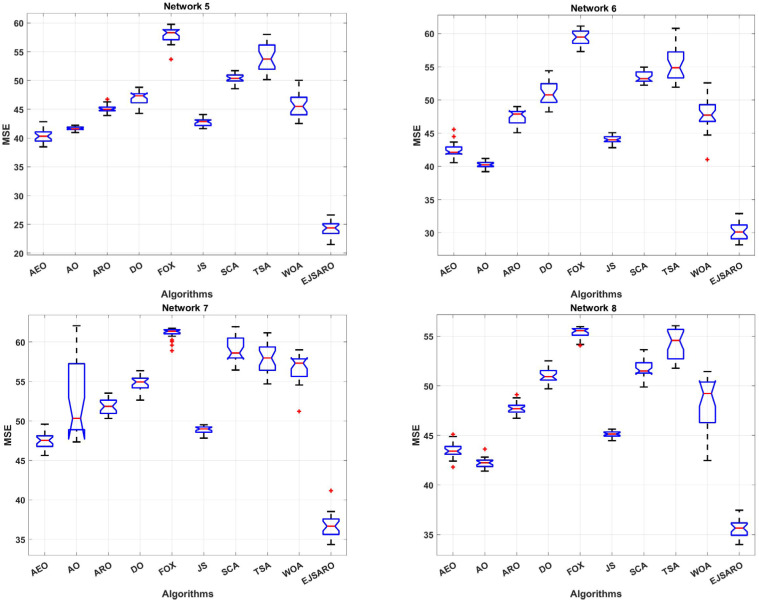
The box plot diagrams of the MSE results on Networks 5 to 8 (the red points are the outliers).

**Figure 12:**
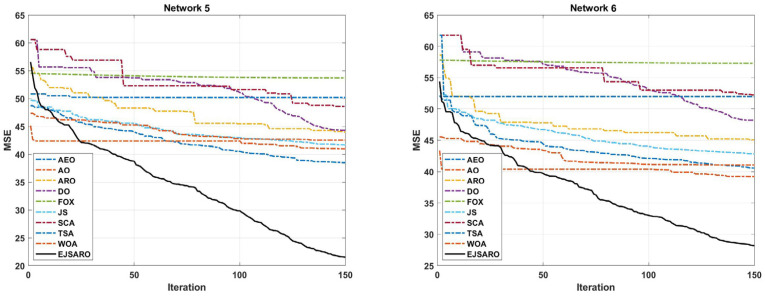

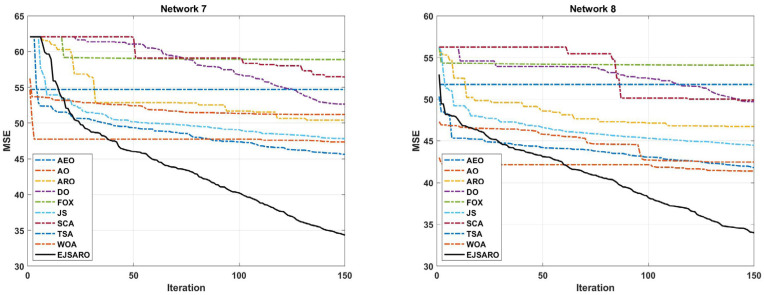
The best convergence of the algorithms in Networks 5 to 8.

In the second set of experiments, the effect of the number of unknown devices on the efficiency of positioning algorithms has been investigated. In this regard, four more three-dimensional IoT networks, Networks 5 to 8, have been designed, in which 150, 200, 250, and 300 IoT devices with unknown positions are deployed. In these networks, the deployment environment is set to $200\times 200\times 150\;m^{3}$, and the communication range is considered 50. Likewise, 40 beacons have been placed in these networks. The statistical results of the algorithms regarding the MSE are given in Table 5. To provide a more detailed comparison, the box plot diagrams and the best convergence of the algorithms on Networks 5 to 8 are plotted in Figure 11 and Figure 12, respectively.

Regarding the numerical results of Table 5, it can be observed that the EJSARO algorithm acquired the best performance among the competitors in terms of the MSE metric. In Networks 5 to 8, the best and worst solutions and the average and median of the obtained solutions by the EJSARO are significantly better than the others. Therefore, it can be asserted that the exploration and exploitation capabilities of the EJSARO are better than the AEO, AO, ARO, DO, FOX, JS, SCA, TSA, and WOA algorithms. Regarding the standard deviation, which indicates the coherence of the set of solutions found by algorithms, other algorithms have better STD than the EJSARO. Nonetheless, by examining the box plot diagrams depicted in Figure 11, it is obvious that the set of solutions reached by the EJSARO is much better than the others and fluctuates in lower MSE values. It is worth mentioning that the AO algorithm took the second-best in most of the experiments conducted on Networks 5 to 8. Also, the box plots demonstrate that the range of solutions achieved by the EJSARO is significantly better than other compared algorithms regarding the MSE.

**Figure 13:**
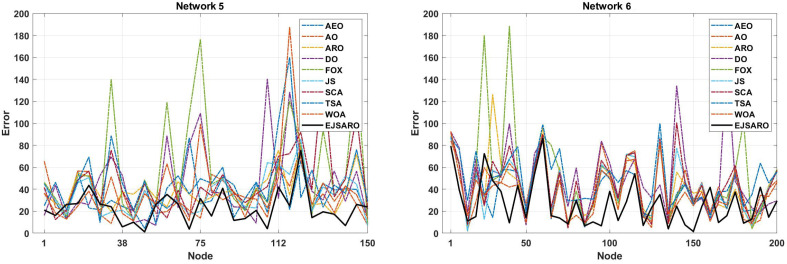

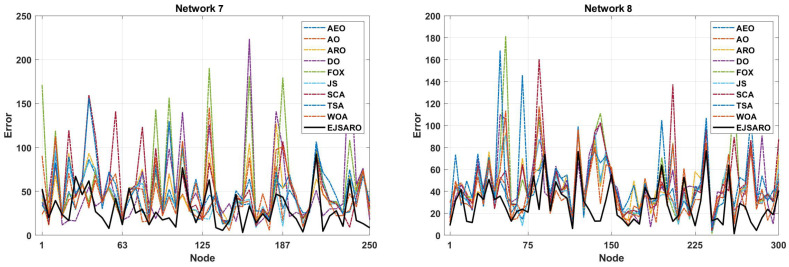
The NLE of algorithms in Networks 5 to 8.

According to the convergence curves illustrated in Figure 12, the EJSARO has an extraordinary convergence rate compared to competing algorithms. The convergence rate of the EJSARO algorithm in Networks 5 to 8 is continuous and sharp, which exhibits its excellent exploration and exploitation capabilities and the appropriate balance between them. It is also evident that the EJSARO algorithm does not have an MSE in successive iterations, which indicates its high ability to escape and not become trapped in local optima. Therefore, it can be concluded that the proposed algorithm has performed well in generating diverse solutions, trying to escape the trap of local optimality by generating new solutions and exploring more of the problem space. In the following, the NLE results of the algorithms on Networks 5 to 8 are provided in Figure 13.

**Figure 14:**
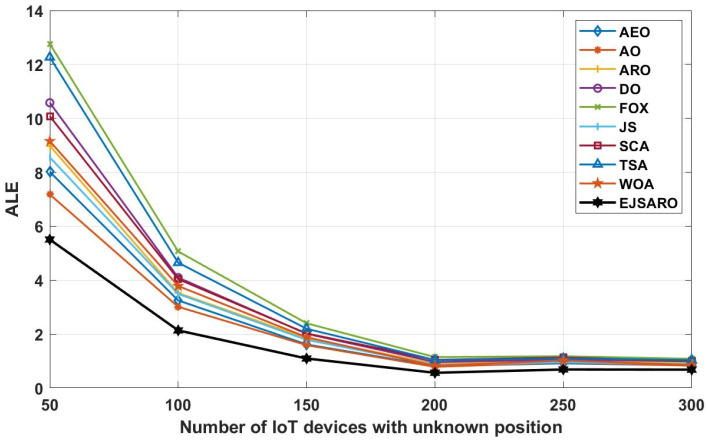
The best ALE values obtained by the algorithms on Networks with different IoT devices.

The NLE line graphs of Figure 13 manifest that the amount of positioning error in EJSARO is much lower than that of AEO, AO, ARO, DO, FOX, JS, SCA, TSA, and WOA algorithms. This means that the proposed algorithm can estimate the position of unknown network objects more accurately, which leads to a reduction in error and an increase in the accuracy of the positioning model. To examine the positioning algorithm more accurately, the best ALE values of the algorithms are presented visually in Figure 14.

**Figure 15:**
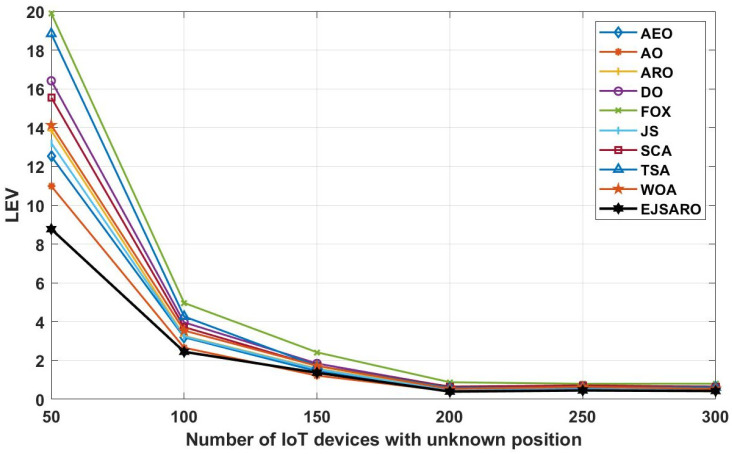
The best LEV values obtained by the algorithms on Networks 5 to 8.

The graphs of Figure 14 reveal that the proposed algorithm was able to achieve better ALE values by making more accurate estimates. Also, these graphs demonstrate that with the increase in the number of network objects, the amount of positioning error decreases, the main reason for which is the rise in the number of hops and the more accurate calculation of the average hop size. To more accurately evaluate the efficiency of the positioning algorithms and compare the set of their predictions, their results are compared in terms of the LEV, and the results of the algorithms on Networks 5 to 8 are plotted in Figure 15.

The curves depicted in Figure 15 indicate that the best solution obtained for Networks 5 to 8, regarding the LEV criterion, belongs to the EJSARO method, except for the network with 150 IoT devices, where the AO algorithm surpassed the competitors. Additionally, the curves illustrated in Figure 15 confirm the results of Figure 14 and assert that the positioning error decreases with the increase in the number of network objects. The main reason for reducing the error by increasing the number of network objects is the more accurate calculation of the average hop size. With the increase in the number of network objects, the objects’ minimum number of hops to the anchors/beacons is calculated more accurately, and as a result, the hop length is estimated with less error, which leads to a more accurate position estimation.

### 5.6. Results on Networks with Different Numbers of Anchors

**Figure 16:**
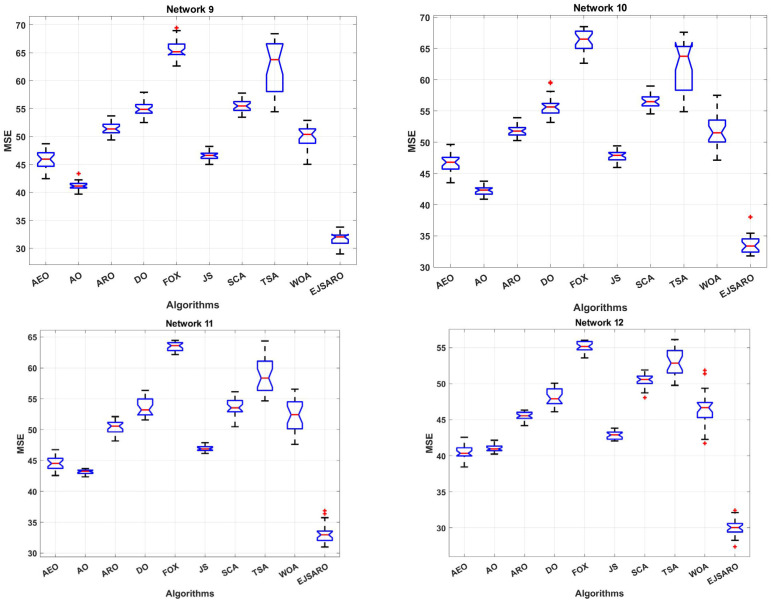
The box plot diagrams of the MSE results on Networks 9 to 12 (the red points are the outliers).

**Figure 17:**
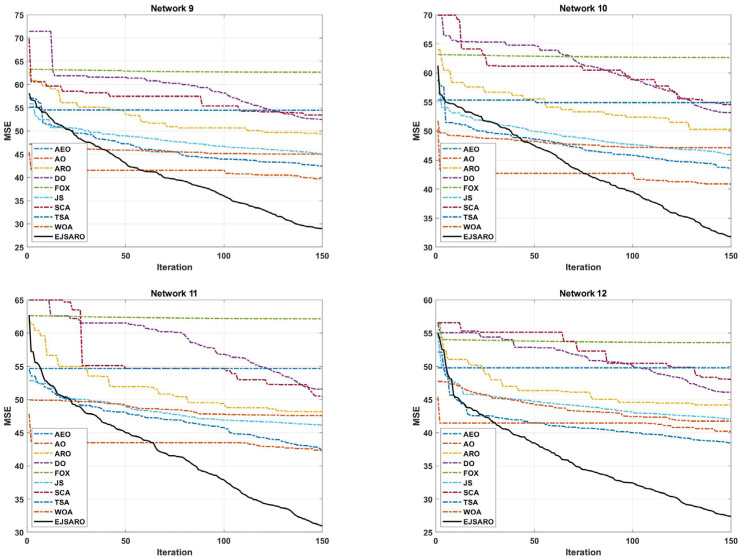
The best convergence of the algorithms in Networks 9 to 12.

This subsection discloses the results of the experiments conducted to evaluate the proficiency of the positioning algorithms in networks with different numbers of anchors/beacons. For this purpose, four 3D IoT networks (labeled Networks 9 to 12) are developed with 30, 35, 40, and 45 location-aware beacons, respectively. Then, the algorithms have been applied to estimate the position of unknown nodes. It is worth mentioning that in these networks, the deployment area is considered $200\times 200\times 150\;m^{3}$, the communication range is fixed to 50 m, and the number of unknown devices is set to 200. The statistical results of the algorithms in terms of MSE are presented in Table 6, box diagrams in Figure 16, and the convergence curves in Figure 17.

The statistical results of Table 6 state that the best and worst MSE values obtained by the EJSARO algorithm are better than similar algorithms, including the AEO, AO, ARO, DO, FOX, JS, SCA, TSA, and WOA algorithms. Also, these results affirm that the proposed algorithm outperforms the competing methods in terms of mean, median, and standard deviation of MSE values.

According to the box diagrams in Figure 16, it is evident that the MSE of the set of predictions made by the EJSARO algorithm is significantly better than the competing algorithms. These diagrams also show that the EJSARO can produce superior solutions in independent executions than similar algorithms, thus proving its higher reliability.

**Figure 18:**
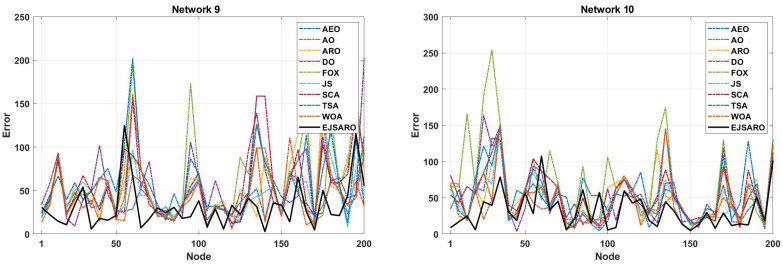

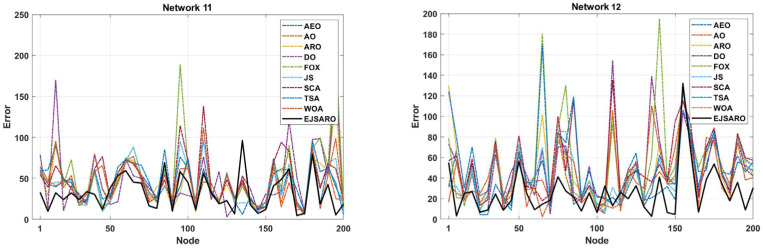
The NLE of the algorithms in Networks 9 to 12.

In terms of convergence rate, the curves of Figure 17 indicate that the proposed algorithm had a consistent convergence and was able to reach a more optimal solution in a shorter period of time, which is due to its high exploration and exploitation capabilities. In addition, the continuous reduction in the convergence curve of the EJSARO method demonstrates that this algorithm did not fall into the trap of local optimums or was able to get out of them in an adequate way. For a more detailed comparison of the algorithms, the NLE values are provided visually in Figure 18.

**Figure 19:**
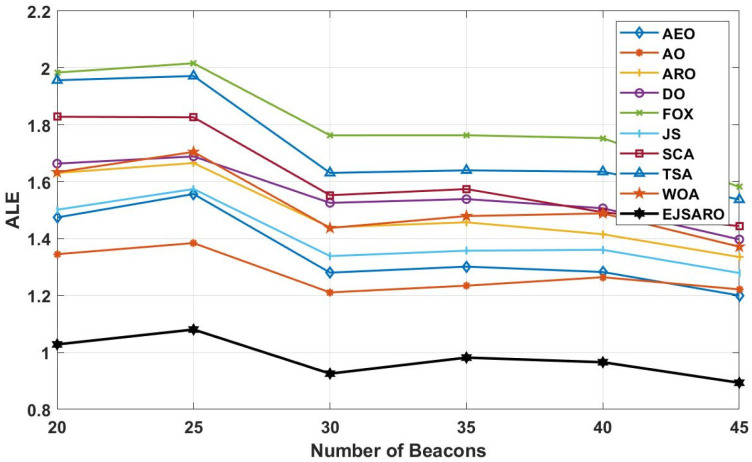
The best ALE values obtained by the algorithms on Networks 9 to 12.

The deep examination of NLE curves illustrated in Figure 18 indicates that the EJSARO has achieved a lower error rate in almost most of the nodes in Networks 9 to 12. In some nodes, the proposed model could not achieve a good error rate. However, considering that the EJSARO model has a good ability to cover more than 90% of the nodes in reducing the error rate, the poor performance in some nodes can be ignored. In the following, the positioning models are compared regarding the ALE, and the results are presented in Figure 19.

**Figure 20:**
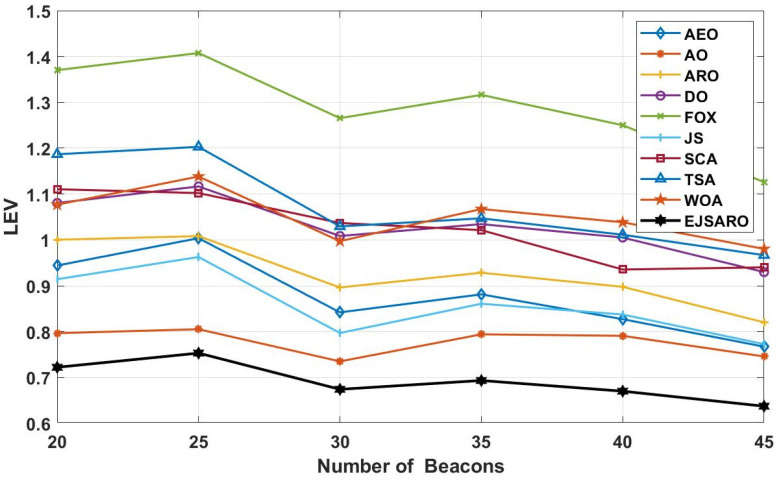
The best LEV values obtained by the algorithms on Networks 9 to 12.

According to the ALE results provided in Figure 19, the proposed model has performed significantly better than other meta-heuristic-based localization models in this series of experiments conducted on Networks 9 to 12. In these networks, the AO took second place regarding the ALE evaluation metric. The huge difference between the results of the proposed algorithm and other algorithms indicates its higher capabilities in estimating the position of the network’s unknown objects in different conditions. The FOX algorithm has achieved the worst results in these networks regarding the ALE and has performed poorly compared to other positioning algorithms. Also, the curves of Figure 19 assert that the ALE values reduce by increasing the number of anchors/beacons in the networks. In the last part of the third set of experiments, Figure 20 provides the LEV results of the algorithms on Networks 9 to 12.

Considering the LEV results reported in Figure 20, the EJSARO algorithm has achieved better results than AEO, AO, ARO, DO, FOX, JS, SCA, TSA, and WOA algorithms and has surpassed them. In these networks, the worst performance is related to the FOX algorithm, which has taken last place. In these graphs, it is clear that the best predictions obtained by the EJSARO algorithm for Networks 9 to 12 are enormously better than the rest of the algorithms. In addition, these graphs reveal that with the increase in the number of anchors/beacons, the average accuracy of the models increases, the main reason for which is the generation of more equations in Equation (35), which leads to more precise initial position estimation by Equation (40).

### 5.7. Networks with Different Communication Range

**Figure 21:**
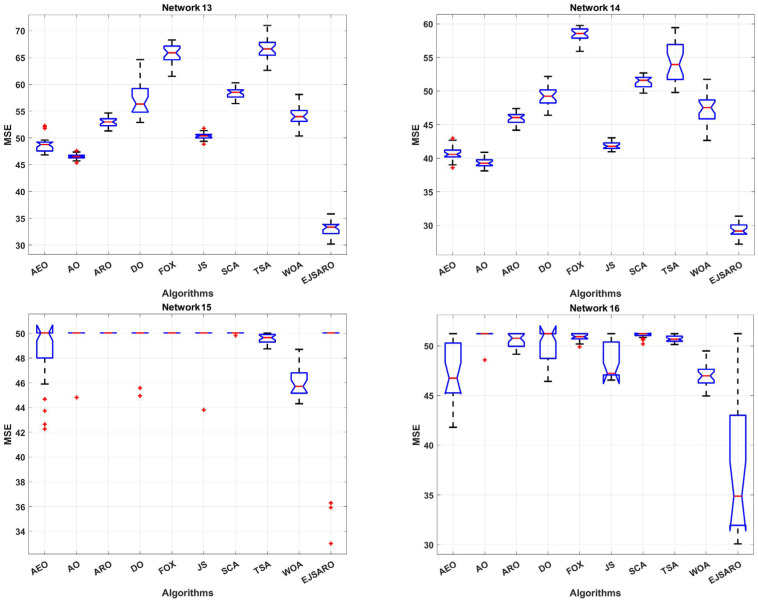
The box plot diagrams of the MSE results on Networks 13 to 16 (the red points are the outliers).

**Figure 22:**
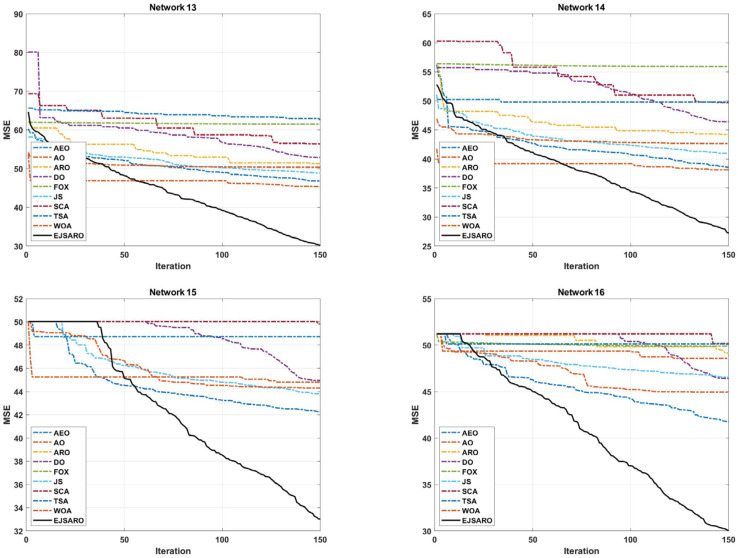
The best convergence of the algorithms in Networks 13 to 16.

The DV-Hop and the proposed algorithm are a range-free method and are able to locate objects with any communication range. Nonetheless, the communication range affects the hop-counting and distance estimation process. Consequently, four more three-dimensional IoT networks, in which the devices have different communication ranges, are designed in this subsection. The communication range is set to 45, 50, 55, and 60 m in Networks 13 to 16, respectively. Also, the network size, number of unknown IoT devices, and beacon count are equal to $200\times 200\times 150\;m^{3}$, 200, and 40, respectively. The statistical results of the positioning algorithms obtained on Network 13 to Network 16 in exposed in Table 7. Likewise, Figure 21 demonstrates the box plots of the results of algorithms, and Figure 22 compares the convergence rate of algorithms in these networks.

The statistical results of Table 7 and the box diagrams of Figure 21 express that the EJSARO algorithm outperformed its competitors in terms of best, worst, mean, median, and standard deviation of MSE and achieved better results. Additionally, the results exhibit that the set of estimations made by the EJSARO algorithm in independent executions is at much lower levels of MSE, which reveals its superiority and remarkable search capabilities. However, the worst, median, and average MSE values obtained by the WOA algorithm on Network 15 are better than other algorithms. Nevertheless, the best solution for this network is still found by the proposed method. In Network 16, the worst solution found by the WOA algorithm is better than other algorithms, but the EJSARO algorithm has surpassed its competitors in terms of statistical criteria and reached a lower MSE.

**Figure 23:**
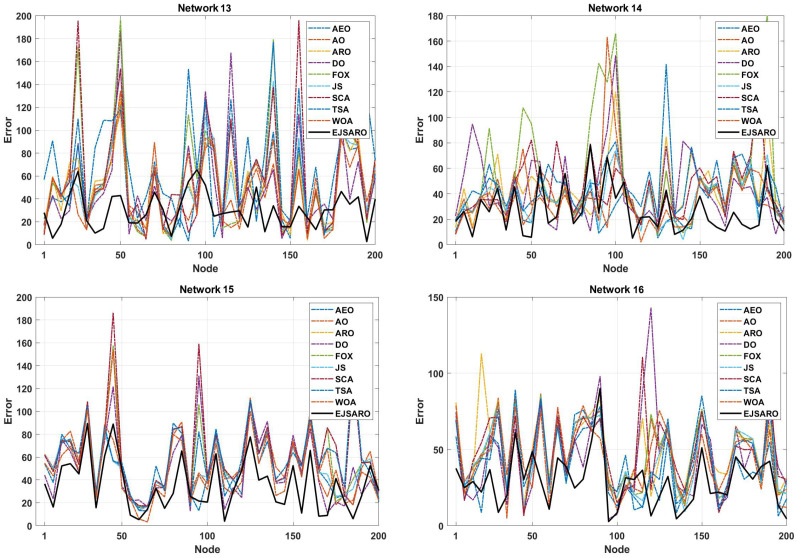
The NLE of the algorithms in Networks 13 to 16.

The convergence curves of Figure 22 indicate that the best solutions for Networks 13 to 16 have been found by the EJSARO. Additionally, these curves represent that the convergence rate of the proposed method is significantly better than the convergence rate of AEO, AO, ARO, DO, FOX, JS, SCA, TSA, and WOA algorithms. Consequently, it can be concluded that the EJSARO has been able to overcome its competitors and provide more optimal solutions in less time. Similarly, the NLE values of the algorithms are presented in Figure 23 to provide a more in-depth comparison.

**Figure 24:**
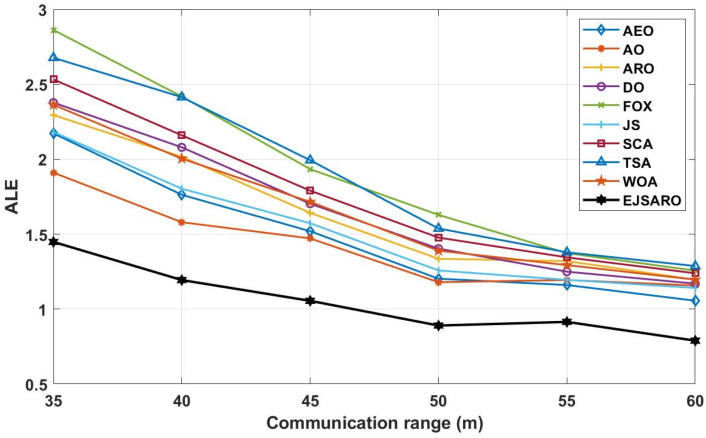
The best ALE values obtained by the algorithms on Networks 13 to 16.

The NLE diagrams of Figure 23 show that the localization error rate in the EJSARO method is lower than in similar algorithms. It is also clear in these graphs that the proposed method has been able to calculate the position of individual nodes more appropriately and achieve a lower average localization error. In the following, the results of the algorithms are compared by the ALE criterion to provide a more accurate evaluation; the comparison results are presented graphically in Figure 24.

**Figure 25:**
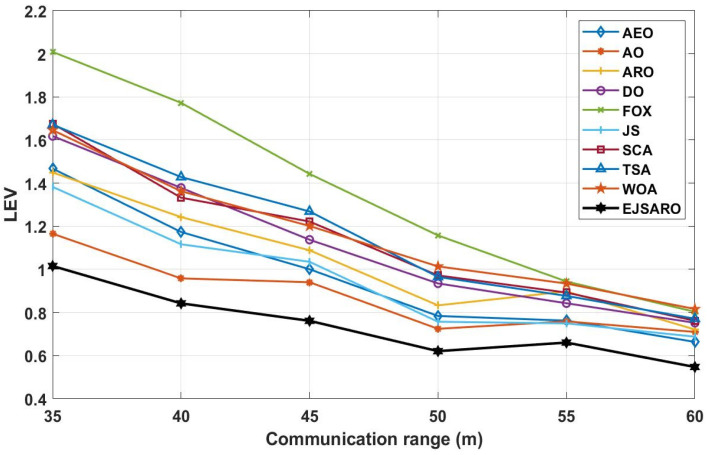
The best LEV values obtained by the algorithms on Networks 13 to 16.

According to the ALE results reported in Figure 24, it is noticeable that the EJSARO method performed better than the competing algorithms and reached lower ALE values, which indicates that the proposed algorithm is capable of accurate calculation of the location of unknown objects in the network through its unique capabilities. As a result, considering that the proposed algorithm has reached the optimal results in Networks 13 to 16 regarding the ALE values, it can be claimed that the EJSARO algorithm has also surpassed its competitors. In addition, the graphs of Figure 24 reveal that the communication range has a notable impact on the efficiency of the models, and the accuracy of the models increases with the increase in the communication range. The major reason for this is to obtain the minimum hop counts more precisely by increasing the R. Figure 25 also compares the results of the algorithms on Networks 13 to 16 visually regarding the LEV metric.

The diagrams in Figure 25 declare that the EJSARO has reached more optimal values regarding the LEV criteria in Networks 13 to 16 and outperformed its competitors, including AEO, AO, ARO, DO, FOX, JS, SCA, TSA, and WOA algorithms. Also, the graphs of Figure 25, in addition to indicating the superiority of EJSARO over its competitors, confirm the results of Figure 24 and reveal that the error of the models decreases with the increase in the communication range, the main reason for which was explained earlier.

### 5.8. Quantitative Performance Evaluation

To provide a clear and quantitative measure of the proposed framework’s effectiveness, in this subsection, a quantitative evaluation is presented to rigorously demonstrate the effectiveness of the proposed framework. To achieve this, the relative improvement of the EJSARO algorithm in comparison to competitor methods was carefully computed and analyzed. The relative improvement ratio (RI) for each comparative method is calculated as follows:

(58) $$RI\left(\%\right)=\frac{{MSE}_{competitor}-\;{MSE}_{EJSARO}}{{MSE}_{competitor}}\times 100$$

where ${MSE}_{competitor}$ denotes the mean square error value obtained by a baseline algorithm, and ${MSE}_{EJSARO}$ represents the corresponding result achieved by the proposed method. This ratio expresses how much EJSARO reduces the positioning error relative to each benchmark method. A higher RI value indicates a more significant accuracy improvement.

Table 8 presents the improvement ratios of the EJSARO over nine state-of-the-art localization algorithms across sixteen 3D IoT environments. It is noteworthy that the RI is calculated by the best MSE values obtained by the algorithms.

As can be observed from Table 8, the EJSARO consistently achieves a high positive RI value across various experimental conditions. This consistent achievement of a high positive RI value is a strong indicator of its higher accuracy in large-scale 3D localization tasks.

## 6. Discussion

Extensive experimentation was undertaken, focusing on a set of sixteen distinct three-dimensional IoT networks, each characterized by unique specifications and configurations. We rigorously analyzed the comprehensive collection of numerical and visual data generated by these experiments. To benchmark the performance of the proposed EJSARO method, a multitude of comparisons were performed against several state-of-the-art techniques. The comparative analysis included the assessment of algorithms such as AEO, AO, ARO, DO, FOX, JS, SCA, TSA, and WOA. The evaluation process considered a diverse range of criteria, specifically MSE (Mean Squared Error), NLE (Normalized Localization Error), ALE (Average Localization Error), and LEV (Localization Error Variance). Based on the thorough examination of both the numerical outcomes and visual representations derived from these experiments, as well as the extensive comparative studies conducted across the aforementioned evaluation metrics, a definitive conclusion can be drawn.

The higher performance of our proposed EJSARO algorithm can be attributed to the complementary hybridization of ARO and JS. In this integration, the ARO provides strong global exploration during early iterations, while the JS enhances local exploitation in later phases. Also, the Lévy flight perturbation provides occasional long and short jumps, which prevent premature convergence and local optima. Moreover, the local search phase fine-tunes the best search agent to further reduce the MSE. The combination of these mechanisms results in a strong optimization approach that is capable of optimizing the nonlinear and multimodal nature of 3D localization problems.

Furthermore, the relative improvement ratio test is conducted based on the MSE values obtained by the algorithms on sixteen 3D IoT environments. The results reveal that the proposed EJSARO algorithm achieves accuracy improvements of 30.38%, 29.78%, 38.26%, 41.78%, 50.90%, 34.98%, 43.72%, 45.54%, and 35.65% over the AEO, AO, ARO, DO, FOX, JS, SCA, TSA, and WOA algorithms, respectively. These quantitative improvements provide evidence that reveals the effectiveness of the combination of ARO, JS, Lévy flight, and the local search mechanism within the EJSARO.

In some low-complexity test environments, the WOA marginally outperformed EJSARO. This behavior is observed in test environments with smooth fitness surfaces and low-dimensional search spaces, where WOA’s encircling and spiral movements can achieve fast convergence without much need for exploration. In the more challenging 3D localization scenarios, the WOA tends to lose diversity and become trapped in local optima, whereas the EJSARO maintains high stability and accuracy due to its adaptive exploration and exploitation capabilities. Overall, the combination of JS, ARO, Lévy flight, and local search strategy enables EJSARO to deliver a consistent and significant improvement in 3D IoT localization accuracy compared to other existing metaheuristic-based positioning models.

## 7. Conclusions and Future Directions

The current paper proposes an innovative positioning framework named EJSARO for three-dimensional Internet of Things (3D IoT) environments. The proposed framework is range-free and requires no additional equipment, such as the Global Positioning System (GPS). In the initial step of the EJSARO, the original Distance Vector-Hop (DV-Hop) is extended to be applicable in 3D IoT networks and to count the hops between network devices. The 3D-DV-Hop is then combined with the multilateration method to estimate the initial position of unknown IoT devices. In the second phase, the Jellyfish Search (JS) and Artificial Rabbits Optimization (ARO) algorithms are modified and lightened. Following this, the modified JS and ARO are hybridized in a complementary manner. The resulting hybrid algorithm is enhanced with a Levy-flight-based intermediate phase. Since the best search agent has a remarkable impact on population guiding and the final result, a local search mechanism is also introduced to exploit the so-far found best search agent. The hybrid meta-heuristic algorithm is then employed to enhance the initially estimated positions.

To assess the potency of the EJSARO, sixteen three-dimensional IoT networks with different specifications are designed. The networks are organized into four categories regarding the varying parameters. The EJSARO is then applied to the networks to detect the position of unknown IoT devices. The results of the EJSARO are compared with the 3D-DV-Hop-based AEO, AO, ARO, DO, FOX, JS, SCA, TSA, and WOA algorithms numerically and visually in terms of Mean Squared Error (MSE), Node Localization Error (NLE), Average Localization Error (ALE), and Localization Error Variance (LEV). In performance evaluations, the results of algorithms are compared by the statistical metrics, including the best and worst achieved solutions and the average, median, and standard deviation of the obtained set of solutions. Likewise, the results are compared using the box plot diagrams and convergence rate curves. The impact of the network’s parameters is also investigated and discussed.

For future studies, the impact of other range-free or range-based methods in the initialization phase can be investigated. The proposed framework can also be adapted for mobile IoT and EC environments.

## Figures and Tables

**Table 1:** Running environment specifications.

Name	Value
Hardware	
CPU	AMD Ryzen 7 3800XT
Frequency	3.9 GHz
RAM	32 GB
Hard drive	1 TB m.2
Software	
Operating system	Windows 11
Language	MATLAB R2021a

**Table 2:** Parameter values of the proposed and competitor algorithms.

Parameter	Value									
	AEO	AO	ARO	DO	FOX	JS	SCA	TSA	WOA	EJSARO
N	30	30	30	30	30	30	30	30	30	30
mi	150	150	150	150	150	150	150	150	150	150
$r_{i}$	[0,1]	-	[0,1]	-	-	-	[0,1]	-	[0,1]	[0,1]
h	2 × rand-1	-	-	-	-	-	-	-	-	-
δ	-	0.1	-	-	-	-	-	-	-	-
ω	-	0.005	-	-	-	-	-	-	-	-
α	-	0.1	-	[0,1]	-	-	-	-	[0,2]	0.05
$n_{i}$	-	-	N(0,1)		-	-	-	-	-	N(0,1)
k	-	-	-	[0,1]	-	-	-	-	-	-
$c_{1}$	-	-	-	-	0.18	-	-	-	-	-
$c_{2}$	-	-	-	-	0.82	-	-	-	-	-
p	-	-	-	-	[0,1]	-	-	-	[0,1]	-
${LV}_{0}$	-	-	-	-	-	0.3	-	-	-	-
β	-	-	-	-	-	3	-	-	-	-
γ	-	-	-	-	-	0.1	-	-	-	-
η	-	-	-	-	-	4	-	-	-	-
$X_{min}$	-	-	-	-	-	-	-	1	-	-
$X_{max}$	-	-	-	-	-	-	-	4	-	-
A	-	-	-	-	-	-	-	-	[−1,1]	-
l	-	-	-	-	-	-	-	-	[−1,1]	-
$\sigma_{\gamma }$	-	-	-	-	-	-	-	-	-	1
ϑ	-	-	-	-	-	-	-	-	-	1.5

**Table 3:** Characteristics of IoT networks.

Parameter	Value
Deployment area	200×200×150~ $500\times 500\times 150\;m^{3}$
Unknown objects	50~300
Anchors	20~50
Communication range	35~70 m

**Table 4:** The statistical results of the algorithms obtained on Networks 1 to 4 regarding the MSE.

Network	Algorithm	Metric				
		Best	Worst	Average	Median	STD
1	AEO	8.0585$\;\times 10^{1}$	8.6818$\;\times 10^{1}$	8.2986$\;\times 10^{1}$	8.2825$\;\times 10^{1}$	1.6606$\;\times 10^{0}$
	AO	8.4847$\;\times 10^{1}$	8.7196$\;\times 10^{1}$	8.6062$\;\times 10^{1}$	8.6126$\;\times 10^{1}$	7.2265$\;\times 10^{-1}$
	ARO	8.6241$\;\times 10^{1}$	9.2784$\;\times 10^{1}$	8.9967$\;\times 10^{1}$	8.9988$\;\times 10^{1}$	1.5948$\;\times 10^{0}$
	DO	8.9024$\;\times 10^{1}$	9.9398$\;\times 10^{1}$	9.5677$\;\times 10^{1}$	9.6405$\;\times 10^{1}$	2.8071$\;\times 10^{0}$
	FOX	1.0846$\;\times 10^{2}$	1.1484$\;\times 10^{2}$	1.1201$\;\times 10^{2}$	1.1193$\;\times 10^{2}$	1.8338$\;\times 10^{0}$
	JS	8.5580$\;\times 10^{1}$	8.9752$\;\times 10^{1}$	8.7730$\;\times 10^{1}$	8.7637$\;\times 10^{1}$	1.0845$\;\times 10^{0}$
	SCA	9.5876$\;\times 10^{1}$	9.9477$\;\times 10^{1}$	9.7493$\;\times 10^{1}$	9.7462$\;\times 10^{1}$	1.0312$\;\times 10^{0}$
	TSA	9.7248$\;\times 10^{1}$	1.0970$\;\times 10^{2}$	1.0330$\;\times 10^{2}$	1.0233$\;\times 10^{2}$	3.4254$\;\times 10^{0}$
	WOA	8.7557$\;\times 10^{1}$	9.6001$\;\times 10^{1}$	9.0876$\;\times 10^{1}$	9.1085$\;\times 10^{1}$	2.2366$\;\times 10^{0}$
	EJSARO	5.1540$\;\times 10^{1}$	6.0936$\;\times 10^{1}$	5.5748$\;\times 10^{1}$	5.5388$\;\times 10^{1}$	2.3553$\;\times 10^{0}$
2	AEO	6.9377$\;\times 10^{1}$	7.6163$\;\times 10^{1}$	7.3226$\;\times 10^{1}$	7.3280$\;\times 10^{1}$	2.0042$\;\times 10^{0}$
	AO	7.2244$\;\times 10^{1}$	7.6085$\;\times 10^{1}$	7.3338$\;\times 10^{1}$	7.3285$\;\times 10^{1}$	8.8176$\;\times 10^{-1}$
	ARO	7.8814$\;\times 10^{1}$	8.4435$\;\times 10^{1}$	8.1709$\;\times 10^{1}$	8.1479$\;\times 10^{1}$	1.7310$\;\times 10^{0}$
	DO	8.4884$\;\times 10^{1}$	9.3859$\;\times 10^{1}$	8.8723$\;\times 10^{1}$	8.8252$\;\times 10^{1}$	2.3001$\;\times 10^{0}$
	FOX	1.0031$\;\times 10^{2}$	1.0632$\;\times 10^{2}$	1.0451$\;\times 10^{2}$	1.0492$\;\times 10^{2}$	1.6625$\;\times 10^{0}$
	JS	7.4538$\;\times 10^{1}$	7.9370$\;\times 10^{1}$	7.6824$\;\times 10^{1}$	7.6784$\;\times 10^{1}$	1.1456$\;\times 10^{0}$
	SCA	8.8991$\;\times 10^{1}$	9.4410$\;\times 10^{1}$	9.1388$\;\times 10^{1}$	9.1274$\;\times 10^{1}$	1.4154$\;\times 10^{0}$
	TSA	8.9304$\;\times 10^{1}$	1.0496$\;\times 10^{2}$	9.7147$\;\times 10^{1}$	9.7595$\;\times 10^{1}$	4.9815$\;\times 10^{0}$
	WOA	7.8792$\;\times 10^{1}$	9.3250$\;\times 10^{1}$	8.5961$\;\times 10^{1}$	8.6540$\;\times 10^{1}$	4.0624$\;\times 10^{0}$
	EJSARO	4.5457$\;\times 10^{1}$	5.3666$\;\times 10^{1}$	4.9956$\;\times 10^{1}$	5.0296$\;\times 10^{1}$	2.1965$\;\times 10^{0}$
3	AEO	1.0086$\;\times 10^{2}$	1.1723$\;\times 10^{2}$	1.0925$\;\times 10^{2}$	1.0833$\;\times 10^{2}$	4.5157$\;\times 10^{0}$
	AO	9.5224$\;\times 10^{1}$	9.9273$\;\times 10^{1}$	9.7356$\;\times 10^{1}$	9.7302$\;\times 10^{1}$	1.2105$\;\times 10^{0}$
	ARO	1.1368$\;\times 10^{2}$	1.2324$\;\times 10^{2}$	1.1941$\;\times 10^{2}$	1.1944$\;\times 10^{2}$	2.2609$\;\times 10^{0}$
	DO	1.1927$\;\times 10^{2}$	1.4957$\;\times 10^{2}$	1.3409$\;\times 10^{2}$	1.3384$\;\times 10^{2}$	8.7404$\;\times 10^{0}$
	FOX	1.4951$\;\times 10^{2}$	1.6789$\;\times 10^{2}$	1.6068$\;\times 10^{2}$	1.6153$\;\times 10^{2}$	5.0209$\;\times 10^{0}$
	JS	1.0542$\;\times 10^{2}$	1.1324$\;\times 10^{2}$	1.0924$\;\times 10^{2}$	1.0884$\;\times 10^{2}$	2.1089$\;\times 10^{0}$
	SCA	1.2413$\;\times 10^{2}$	1.3447$\;\times 10^{2}$	1.2870$\;\times 10^{2}$	1.2817$\;\times 10^{2}$	2.5658$\;\times 10^{0}$
	TSA	1.3117$\;\times 10^{2}$	1.5445$\;\times 10^{2}$	1.4169$\;\times 10^{2}$	1.4201$\;\times 10^{2}$	5.3133$\;\times 10^{0}$
	WOA	1.0666$\;\times 10^{2}$	1.3173$\;\times 10^{2}$	1.1769$\;\times 10^{2}$	1.1783$\;\times 10^{2}$	6.3694$\;\times 10^{0}$
	EJSARO	6.8457$\;\times 10^{1}$	8.0358$\;\times 10^{1}$	7.3043$\;\times 10^{1}$	7.2841$\;\times 10^{1}$	2.9991$\;\times 10^{0}$
4	AEO	1.1379$\;\times 10^{2}$	1.3513$\;\times 10^{2}$	1.2562$\;\times 10^{2}$	1.2687$\;\times 10^{2}$	5.7722$\;\times 10^{0}$
	AO	1.0593$\;\times 10^{2}$	1.1062$\;\times 10^{2}$	1.0854$\;\times 10^{2}$	1.0861$\;\times 10^{2}$	1.0922$\;\times 10^{0}$
	ARO	1.2877$\;\times 10^{2}$	1.4068$\;\times 10^{2}$	1.3586$\;\times 10^{2}$	1.3661$\;\times 10^{2}$	3.2653$\;\times 10^{0}$
	DO	1.4729$\;\times 10^{2}$	1.8186$\;\times 10^{2}$	1.6539$\;\times 10^{2}$	1.6603$\;\times 10^{2}$	8.7579$\;\times 10^{0}$
	FOX	1.9110$\;\times 10^{2}$	2.0885$\;\times 10^{2}$	1.9978$\;\times 10^{2}$	1.9981$\;\times 10^{2}$	4.8409$\;\times 10^{0}$
	JS	1.2884$\;\times 10^{2}$	1.3948$\;\times 10^{2}$	1.3498$\;\times 10^{2}$	1.3536$\;\times 10^{2}$	2.4003$\;\times 10^{0}$
	SCA	1.4079$\;\times 10^{2}$	1.5389$\;\times 10^{2}$	1.4671$\;\times 10^{2}$	1.4620$\;\times 10^{2}$	3.5883$\;\times 10^{0}$
	TSA	1.6413$\;\times 10^{2}$	1.9812$\;\times 10^{2}$	1.7816$\;\times 10^{2}$	1.7590$\;\times 10^{2}$	9.5714$\;\times 10^{0}$
	WOA	1.2864$\;\times 10^{2}$	1.6104$\;\times 10^{2}$	1.4618$\;\times 10^{2}$	1.4608$\;\times 10^{2}$	9.9216$\;\times 10^{0}$
	EJSARO	8.0525$\;\times 10^{1}$	9.7423$\;\times 10^{1}$	8.9890$\;\times 10^{1}$	8.8791$\;\times 10^{1}$	4.0554$\;\times 10^{0}$

**Table 5:** The statistical results of the algorithms obtained on Networks 5 to 8 regarding the MSE.

Network	Algorithm	Metric				
		Best	Worst	Average	Median	STD
5	AEO	3.8477$\;\times 10^{1}$	4.2828$\;\times 10^{1}$	4.0411$\;\times 10^{1}$	4.0325$\;\times 10^{1}$	1.1801$\;\times 10^{0}$
	AO	4.0965$\;\times 10^{1}$	4.2210$\;\times 10^{1}$	4.1665$\;\times 10^{1}$	4.1592$\;\times 10^{1}$	**3.4345** $\;\times 10^{-1}$
	ARO	4.3929$\;\times 10^{1}$	4.6758$\;\times 10^{1}$	4.5101$\;\times 10^{1}$	4.5006$\;\times 10^{1}$	6.6883$\;\times 10^{-1}$
	DO	4.4288$\;\times 10^{1}$	4.8812$\;\times 10^{1}$	4.6851$\;\times 10^{1}$	4.7349$\;\times 10^{1}$	1.2132$\;\times 10^{0}$
	FOX	5.3696$\;\times 10^{1}$	5.9774$\;\times 10^{1}$	5.7935$\;\times 10^{1}$	5.8311$\;\times 10^{1}$	1.3649$\;\times 10^{0}$
	JS	4.1640$\;\times 10^{1}$	4.4095$\;\times 10^{1}$	4.2775$\;\times 10^{1}$	4.2861$\;\times 10^{1}$	7.0211$\;\times 10^{-1}$
	SCA	4.8573$\;\times 10^{1}$	5.1701$\;\times 10^{1}$	5.0417$\;\times 10^{1}$	5.0390$\;\times 10^{1}$	7.9788$\;\times 10^{-1}$
	TSA	5.0167$\;\times 10^{1}$	5.8007$\;\times 10^{1}$	5.4054$\;\times 10^{1}$	5.3732$\;\times 10^{1}$	2.5297$\;\times 10^{0}$
	WOA	4.2518$\;\times 10^{1}$	5.0048$\;\times 10^{1}$	4.5626$\;\times 10^{1}$	4.5498$\;\times 10^{1}$	1.9802$\;\times 10^{0}$
	**EJSARO**	**2.1508** $\;\times 10^{1}$	**2.6626** $\;\times 10^{1}$	**2.4356** $\;\times 10^{1}$	**2.4399** $\;\times 10^{1}$	1.3001$\;\times 10^{0}$
6	AEO	4.0567$\;\times 10^{1}$	4.5562$\;\times 10^{1}$	4.2447$\;\times 10^{1}$	4.2144$\;\times 10^{1}$	1.0957$\;\times 10^{0}$
	AO	3.9209$\;\times 10^{1}$	4.1191$\;\times 10^{1}$	4.0226$\;\times 10^{1}$	4.0252$\;\times 10^{1}$	**5.4035** $\;\times 10^{-1}$
	ARO	4.5066$\;\times 10^{1}$	4.9008$\;\times 10^{1}$	4.7554$\;\times 10^{1}$	4.7873$\;\times 10^{1}$	1.0760$\;\times 10^{0}$
	DO	4.8203$\;\times 10^{1}$	5.4398$\;\times 10^{1}$	5.1165$\;\times 10^{1}$	5.0772$\;\times 10^{1}$	1.6538$\;\times 10^{0}$
	FOX	5.7301$\;\times 10^{1}$	6.1139$\;\times 10^{1}$	5.9394$\;\times 10^{1}$	5.9485$\;\times 10^{1}$	1.0112$\;\times 10^{0}$
	JS	4.2815$\;\times 10^{1}$	4.5065$\;\times 10^{1}$	4.4029$\;\times 10^{1}$	4.4005$\;\times 10^{1}$	5.8338$\;\times 10^{-1}$
	SCA	5.2237$\;\times 10^{1}$	5.4966$\;\times 10^{1}$	5.3533$\;\times 10^{1}$	5.3229$\;\times 10^{1}$	8.0877$\;\times 10^{-1}$
	TSA	5.1943$\;\times 10^{1}$	6.0789$\;\times 10^{1}$	5.5372$\;\times 10^{1}$	5.4869$\;\times 10^{1}$	2.4524$\;\times 10^{0}$
	WOA	4.1050$\;\times 10^{1}$	5.2576$\;\times 10^{1}$	4.7886$\;\times 10^{1}$	4.7720$\;\times 10^{1}$	2.1983$\;\times 10^{0}$
	**EJSARO**	**2.8181** $\;\times 10^{1}$	**3.2892** $\;\times 10^{1}$	**3.0247** $\;\times 10^{1}$	**3.0115** $\;\times 10^{1}$	1.3246$\;\times 10^{0}$
7	AEO	4.5620$\;\times 10^{1}$	4.9580$\;\times 10^{1}$	4.7472$\;\times 10^{1}$	4.7542$\;\times 10^{1}$	9.8565$\;\times 10^{-1}$
	AO	4.7348$\;\times 10^{1}$	6.2060$\;\times 10^{1}$	5.2674$\;\times 10^{1}$	5.0337$\;\times 10^{1}$	4.6435$\;\times 10^{0}$
	ARO	5.0323$\;\times 10^{1}$	5.3523$\;\times 10^{1}$	5.1766$\;\times 10^{1}$	5.1847$\;\times 10^{1}$	9.5955$\;\times 10^{-1}$
	DO	5.2651$\;\times 10^{1}$	5.6357$\;\times 10^{1}$	5.4732$\;\times 10^{1}$	5.4937$\;\times 10^{1}$	1.0380$\;\times 10^{0}$
	FOX	5.8885$\;\times 10^{1}$	6.1735$\;\times 10^{1}$	6.1106$\;\times 10^{1}$	6.1363$\;\times 10^{1}$	7.0454$\;\times 10^{-1}$
	JS	4.7827$\;\times 10^{1}$	4.9511$\;\times 10^{1}$	4.8875$\;\times 10^{1}$	4.8991$\;\times 10^{1}$	**4.4577** $\;\times 10^{-1}$
	SCA	5.6441$\;\times 10^{1}$	6.1928$\;\times 10^{1}$	5.8933$\;\times 10^{1}$	5.8606$\;\times 10^{1}$	1.5016$\;\times 10^{0}$
	TSA	5.4695$\;\times 10^{1}$	6.1165$\;\times 10^{1}$	5.7885$\;\times 10^{1}$	5.7976$\;\times 10^{1}$	1.9388$\;\times 10^{0}$
	WOA	5.1204$\;\times 10^{1}$	5.9001$\;\times 10^{1}$	5.6660$\;\times 10^{1}$	5.7321$\;\times 10^{1}$	1.6825$\;\times 10^{0}$
	**EJSARO**	**3.4341** $\;\times 10^{1}$	**4.1171** $\;\times 10^{1}$	**3.6693** $\;\times 10^{1}$	**3.6672** $\;\times 10^{1}$	1.4220$\;\times 10^{0}$
8	AEO	4.1812$\;\times 10^{1}$	4.5094$\;\times 10^{1}$	4.3532$\;\times 10^{1}$	4.3416$\;\times 10^{1}$	7.7386$\;\times 10^{-1}$
	AO	4.1398$\;\times 10^{1}$	4.3628$\;\times 10^{1}$	4.2279$\;\times 10^{1}$	4.2247$\;\times 10^{1}$	5.4990$\;\times 10^{-1}$
	ARO	4.6735$\;\times 10^{1}$	4.9104$\;\times 10^{1}$	4.7744$\;\times 10^{1}$	4.7700$\;\times 10^{1}$	5.6494$\;\times 10^{-1}$
	DO	4.9710$\;\times 10^{1}$	5.2520$\;\times 10^{1}$	5.1027$\;\times 10^{1}$	5.0931$\;\times 10^{1}$	7.2138$\;\times 10^{-1}$
	FOX	5.4078$\;\times 10^{1}$	5.5959$\;\times 10^{1}$	5.5391$\;\times 10^{1}$	5.5562$\;\times 10^{1}$	5.5193$\;\times 10^{-1}$
	JS	4.4475$\;\times 10^{1}$	4.5632$\;\times 10^{1}$	4.5140$\;\times 10^{1}$	4.5138$\;\times 10^{1}$	**2.7679** $\;\times 10^{-1}$
	SCA	4.9886$\;\times 10^{1}$	5.3640$\;\times 10^{1}$	5.1647$\;\times 10^{1}$	5.1495$\;\times 10^{1}$	9.3879$\;\times 10^{-1}$
	TSA	5.1778$\;\times 10^{1}$	5.6061$\;\times 10^{1}$	5.4225$\;\times 10^{1}$	5.4574$\;\times 10^{1}$	1.4814$\;\times 10^{0}$
	WOA	4.2470$\;\times 10^{1}$	5.1437$\;\times 10^{1}$	4.8321$\;\times 10^{1}$	4.9223$\;\times 10^{1}$	2.6234$\;\times 10^{0}$
	**EJSARO**	**3.3991** $\;\times 10^{1}$	**3.7446** $\;\times 10^{1}$	**3.5673** $\;\times 10^{1}$	**3.5660** $\;\times 10^{1}$	9.1040$\;\times 10^{-1}$

**Table 6:** The statistical results of the algorithms on Networks 9 to 12 regarding the MSE.

Network	Algorithm	Metric				
		Best	Worst	Average	Median	STD
9	AEO	4.2462$\;\times 10^{1}$	4.8716$\;\times 10^{1}$	4.5940$\;\times 10^{1}$	4.5956$\;\times 10^{1}$	1.6515$\;\times 10^{0}$
	AO	3.9693$\;\times 10^{1}$	4.3346$\;\times 10^{1}$	4.1151$\;\times 10^{1}$	4.1138$\;\times 10^{1}$	8.3518$\;\times 10^{-1}$
	ARO	4.9393$\;\times 10^{1}$	5.3700$\;\times 10^{1}$	5.1437$\;\times 10^{1}$	5.1364$\;\times 10^{1}$	1.0725$\;\times 10^{0}$
	DO	5.2514$\;\times 10^{1}$	5.7927$\;\times 10^{1}$	5.4900$\;\times 10^{1}$	5.4866$\;\times 10^{1}$	1.2041$\;\times 10^{0}$
	FOX	6.2636$\;\times 10^{1}$	6.9480$\;\times 10^{1}$	6.5634$\;\times 10^{1}$	6.5186$\;\times 10^{1}$	1.6248$\;\times 10^{0}$
	JS	4.5013$\;\times 10^{1}$	4.8239$\;\times 10^{1}$	4.6507$\;\times 10^{1}$	4.6632$\;\times 10^{1}$	**8.2805** $\;\times 10^{-1}$
	SCA	5.3462$\;\times 10^{1}$	5.7782$\;\times 10^{1}$	5.5560$\;\times 10^{1}$	5.5479$\;\times 10^{1}$	1.0427$\;\times 10^{0}$
	TSA	5.4446$\;\times 10^{1}$	6.8415$\;\times 10^{1}$	6.2413$\;\times 10^{1}$	6.3772$\;\times 10^{1}$	4.5779$\;\times 10^{0}$
	WOA	4.5035$\;\times 10^{1}$	5.2899$\;\times 10^{1}$	4.9930$\;\times 10^{1}$	5.0388$\;\times 10^{1}$	1.9349$\;\times 10^{0}$
	**EJSARO**	**2.8984** $\;\times 10^{1}$	**3.3790** $\;\times 10^{1}$	**3.1678** $\;\times 10^{1}$	**3.2035** $\;\times 10^{1}$	1.2366$\;\times 10^{0}$
10	AEO	4.3518$\;\times 10^{1}$	4.9645$\;\times 10^{1}$	4.6671$\;\times 10^{1}$	4.6797$\;\times 10^{1}$	1.3566$\;\times 10^{0}$
	AO	4.0877$\;\times 10^{1}$	4.3757$\;\times 10^{1}$	4.2286$\;\times 10^{1}$	4.2339$\;\times 10^{1}$	**7.0172** $\;\times 10^{-1}$
	ARO	5.0273$\;\times 10^{1}$	5.3926$\;\times 10^{1}$	5.1812$\;\times 10^{1}$	5.1785$\;\times 10^{1}$	1.0069$\;\times 10^{0}$
	DO	5.3175$\;\times 10^{1}$	5.9611$\;\times 10^{1}$	5.5730$\;\times 10^{1}$	5.5647$\;\times 10^{1}$	1.6982$\;\times 10^{0}$
	FOX	6.2652$\;\times 10^{1}$	6.8507$\;\times 10^{1}$	6.6340$\;\times 10^{1}$	6.6509$\;\times 10^{1}$	1.6612$\;\times 10^{0}$
	JS	4.5962$\;\times 10^{1}$	4.9420$\;\times 10^{1}$	4.7821$\;\times 10^{1}$	4.7902$\;\times 10^{1}$	8.2935$\;\times 10^{-1}$
	SCA	5.4539$\;\times 10^{1}$	5.8995$\;\times 10^{1}$	5.6538$\;\times 10^{1}$	5.6495$\;\times 10^{1}$	1.0391$\;\times 10^{0}$
	TSA	5.4894$\;\times 10^{1}$	6.7611$\;\times 10^{1}$	6.1881$\;\times 10^{1}$	6.3765$\;\times 10^{1}$	3.9976$\;\times 10^{0}$
	WOA	4.7129$\;\times 10^{1}$	5.7515$\;\times 10^{1}$	5.1867$\;\times 10^{1}$	5.1509$\;\times 10^{1}$	2.5218$\;\times 10^{0}$
	**EJSARO**	**3.1796** $\;\times 10^{1}$	**3.8033** $\;\times 10^{1}$	**3.3548** $\;\times 10^{1}$	**3.3381** $\;\times 10^{1}$	1.4671$\;\times 10^{0}$
11	AEO	4.2570$\;\times 10^{1}$	4.6746$\;\times 10^{1}$	4.4556$\;\times 10^{1}$	4.4525$\;\times 10^{1}$	1.0749$\;\times 10^{0}$
	AO	4.2360$\;\times 10^{1}$	4.3688$\;\times 10^{1}$	4.3133$\;\times 10^{1}$	4.3239$\;\times 10^{1}$	3.8250$\;\times 10^{-1}$
	ARO	4.8176$\;\times 10^{1}$	5.2102$\;\times 10^{1}$	5.0393$\;\times 10^{1}$	5.0558$\;\times 10^{1}$	9.7717$\;\times 10^{-1}$
	DO	5.1560$\;\times 10^{1}$	5.6333$\;\times 10^{1}$	5.3592$\;\times 10^{1}$	5.3193$\;\times 10^{1}$	1.4214$\;\times 10^{0}$
	FOX	6.2143$\;\times 10^{1}$	6.4454$\;\times 10^{1}$	6.3496$\;\times 10^{1}$	6.3593$\;\times 10^{1}$	7.0436$\;\times 10^{-1}$
	JS	4.6119$\;\times 10^{1}$	4.7883$\;\times 10^{1}$	4.6936$\;\times 10^{1}$	4.6888$\;\times 10^{1}$	4.6347$\;\times 10^{-1}$
	SCA	5.0463$\;\times 10^{1}$	5.6124$\;\times 10^{1}$	5.3743$\;\times 10^{1}$	5.3527$\;\times 10^{1}$	1.3145$\;\times 10^{0}$
	TSA	5.4652$\;\times 10^{1}$	6.4362$\;\times 10^{1}$	5.8709$\;\times 10^{1}$	5.8358$\;\times 10^{1}$	2.9555$\;\times 10^{0}$
	WOA	4.7597$\;\times 10^{1}$	5.6543$\;\times 10^{1}$	5.2165$\;\times 10^{1}$	5.2417$\;\times 10^{1}$	2.6861$\;\times 10^{0}$
	**EJSARO**	**3.0983** $\;\times 10^{1}$	**3.6846** $\;\times 10^{1}$	**3.3110** $\;\times 10^{1}$	**3.2966** $\;\times 10^{1}$	1.4970$\;\times 10^{0}$
12	AEO	3.8447$\;\times 10^{1}$	4.2552$\;\times 10^{1}$	4.0538$\;\times 10^{1}$	4.0344$\;\times 10^{1}$	9.0931$\;\times 10^{-1}$
	AO	4.0228$\;\times 10^{1}$	4.2149$\;\times 10^{1}$	4.1022$\;\times 10^{1}$	4.0938$\;\times 10^{1}$	**4.7259** $\;\times 10^{-1}$
	ARO	4.4168$\;\times 10^{1}$	4.6329$\;\times 10^{1}$	4.5500$\;\times 10^{1}$	4.5535$\;\times 10^{1}$	6.0866$\;\times 10^{-1}$
	DO	4.6089$\;\times 10^{1}$	5.0057$\;\times 10^{1}$	4.8087$\;\times 10^{1}$	4.7891$\;\times 10^{1}$	1.1249$\;\times 10^{0}$
	FOX	5.3565$\;\times 10^{1}$	5.6012$\;\times 10^{1}$	5.5190$\;\times 10^{1}$	5.5149$\;\times 10^{1}$	6.6026$\;\times 10^{-1}$
	JS	4.2042$\;\times 10^{1}$	4.3819$\;\times 10^{1}$	4.2833$\;\times 10^{1}$	4.2884$\;\times 10^{1}$	5.0986$\;\times 10^{-1}$
	SCA	4.8035$\;\times 10^{1}$	5.1887$\;\times 10^{1}$	5.0412$\;\times 10^{1}$	5.0581$\;\times 10^{1}$	9.3716$\;\times 10^{-1}$
	TSA	4.9766$\;\times 10^{1}$	5.6114$\;\times 10^{1}$	5.2967$\;\times 10^{1}$	5.2839$\;\times 10^{1}$	1.8469$\;\times 10^{0}$
	WOA	4.1740$\;\times 10^{1}$	5.1838$\;\times 10^{1}$	4.6486$\;\times 10^{1}$	4.6670$\;\times 10^{1}$	2.4945$\;\times 10^{0}$
	**EJSARO**	**2.7378** $\;\times 10^{1}$	**3.2441** $\;\times 10^{1}$	**3.0050** $\;\times 10^{1}$	**3.0032** $\;\times 10^{1}$	1.2228$\;\times 10^{0}$

**Table 7:** The statistical results of algorithms obtained on Networks 13 to 16 regarding the MSE.

Network	Algorithm	Metric				
		Best	Worst	Average	Median	STD
13	AEO	4.6823$\;\times 10^{1}$	5.2249$\;\times 10^{1}$	4.8753$\;\times 10^{1}$	4.8757$\;\times 10^{1}$	1.4817$\;\times 10^{0}$
	AO	4.5368$\;\times 10^{1}$	4.7639$\;\times 10^{1}$	4.6516$\;\times 10^{1}$	4.6528$\;\times 10^{1}$	**4.9205** $\;\times 10^{-1}$
	ARO	5.1308$\;\times 10^{1}$	5.4645$\;\times 10^{1}$	5.2973$\;\times 10^{1}$	5.2988$\;\times 10^{1}$	8.7507$\;\times 10^{-1}$
	DO	5.2879$\;\times 10^{1}$	6.4601$\;\times 10^{1}$	5.7328$\;\times 10^{1}$	5.6313$\;\times 10^{1}$	3.2550$\;\times 10^{0}$
	FOX	6.1514$\;\times 10^{1}$	6.8275$\;\times 10^{1}$	6.5776$\;\times 10^{1}$	6.5889$\;\times 10^{1}$	1.6421$\;\times 10^{0}$
	JS	4.8858$\;\times 10^{1}$	5.1769$\;\times 10^{1}$	5.0347$\;\times 10^{1}$	5.0368$\;\times 10^{1}$	6.5222$\;\times 10^{-1}$
	SCA	5.6397$\;\times 10^{1}$	6.0279$\;\times 10^{1}$	5.8390$\;\times 10^{1}$	5.8507$\;\times 10^{1}$	1.0118$\;\times 10^{0}$
	TSA	6.2602$\;\times 10^{1}$	7.0983$\;\times 10^{1}$	6.6599$\;\times 10^{1}$	6.6602$\;\times 10^{1}$	2.0329$\;\times 10^{0}$
	WOA	5.0364$\;\times 10^{1}$	5.8104$\;\times 10^{1}$	5.4284$\;\times 10^{1}$	5.3978$\;\times 10^{1}$	2.0213$\;\times 10^{0}$
	**EJSARO**	**3.0190** $\;\times 10^{1}$	**3.5819** $\;\times 10^{1}$	**3.3041** $\;\times 10^{1}$	**3.3361** $\;\times 10^{1}$	1.3236$\;\times 10^{0}$
14	AEO	3.8571$\;\times 10^{1}$	4.2975$\;\times 10^{1}$	4.0617$\;\times 10^{1}$	4.0580$\;\times 10^{1}$	1.0217$\;\times 10^{0}$
	AO	3.8120$\;\times 10^{1}$	4.0894$\;\times 10^{1}$	3.9360$\;\times 10^{1}$	3.9267$\;\times 10^{1}$	6.6661$\;\times 10^{-1}$
	ARO	4.4186$\;\times 10^{1}$	4.7405$\;\times 10^{1}$	4.5944$\;\times 10^{1}$	4.6058$\;\times 10^{1}$	8.6783$\;\times 10^{-1}$
	DO	4.6399$\;\times 10^{1}$	5.2188$\;\times 10^{1}$	4.9256$\;\times 10^{1}$	4.9245$\;\times 10^{1}$	1.4822$\;\times 10^{0}$
	FOX	5.5915$\;\times 10^{1}$	5.9757$\;\times 10^{1}$	5.8375$\;\times 10^{1}$	5.8576$\;\times 10^{1}$	9.6625$\;\times 10^{-1}$
	JS	4.0973$\;\times 10^{1}$	4.3051$\;\times 10^{1}$	4.1904$\;\times 10^{1}$	4.1775$\;\times 10^{1}$	**5.8869** $\;\times 10^{-1}$
	SCA	4.9688$\;\times 10^{1}$	5.2697$\;\times 10^{1}$	5.1404$\;\times 10^{1}$	5.1598$\;\times 10^{1}$	8.7234$\;\times 10^{-1}$
	TSA	4.9780$\;\times 10^{1}$	5.9427$\;\times 10^{1}$	5.4307$\;\times 10^{1}$	5.3952$\;\times 10^{1}$	3.1073$\;\times 10^{0}$
	WOA	4.2655$\;\times 10^{1}$	5.1753$\;\times 10^{1}$	4.7372$\;\times 10^{1}$	4.7539$\;\times 10^{1}$	2.2614$\;\times 10^{0}$
	**EJSARO**	**2.7213** $\;\times 10^{1}$	**3.1391** $\;\times 10^{1}$	**2.9329** $\;\times 10^{1}$	**2.9160** $\;\times 10^{1}$	1.1042$\;\times 10^{0}$
15	AEO	4.2270$\;\times 10^{1}$	5.0030$\;\times 10^{1}$	4.8614$\;\times 10^{1}$	5.0030$\;\times 10^{1}$	2.5837$\;\times 10^{0}$
	AO	4.4816$\;\times 10^{1}$	5.0030$\;\times 10^{1}$	4.9821$\;\times 10^{1}$	5.0030$\;\times 10^{1}$	1.0427$\;\times 10^{0}$
	ARO	5.0030$\;\times 10^{1}$	5.0030$\;\times 10^{1}$	5.0030$\;\times 10^{1}$	5.0030$\;\times 10^{1}$	**1.4504** $\;\times 10^{-14}$
	DO	4.4959$\;\times 10^{1}$	5.0030$\;\times 10^{1}$	4.9649$\;\times 10^{1}$	5.0030$\;\times 10^{1}$	1.3210$\;\times 10^{0}$
	FOX	5.0030$\;\times 10^{1}$	5.0030$\;\times 10^{1}$	5.0030$\;\times 10^{1}$	5.0030$\;\times 10^{1}$	**1.4504** $\;\times 10^{-14}$
	JS	4.3812$\;\times 10^{1}$	5.0030$\;\times 10^{1}$	4.9781$\;\times 10^{1}$	5.0030$\;\times 10^{1}$	1.2435$\;\times 10^{0}$
	SCA	4.9815$\;\times 10^{1}$	5.0030$\;\times 10^{1}$	5.0021$\;\times 10^{1}$	5.0030$\;\times 10^{1}$	4.2977$\;\times 10^{-2}$
	TSA	4.8738$\;\times 10^{1}$	5.0005$\;\times 10^{1}$	4.9541$\;\times 10^{1}$	4.9642$\;\times 10^{1}$	3.7737$\;\times 10^{-1}$
	**WOA**	4.4306$\;\times 10^{1}$	**4.8696** $\;\times 10^{1}$	**4.6166** $\;\times 10^{1}$	**4.5716** $\;\times 10^{1}$	1.2510$\;\times 10^{0}$
	**EJSARO**	**3.3010** $\;\times 10^{1}$	5.0030$\;\times 10^{1}$	4.8235$\;\times 10^{1}$	5.0030$\;\times 10^{1}$	4.9865$\;\times 10^{0}$
16	AEO	4.1791$\;\times 10^{1}$	5.1214$\;\times 10^{1}$	4.7335$\;\times 10^{1}$	4.6741$\;\times 10^{1}$	2.8633$\;\times 10^{0}$
	AO	4.8575$\;\times 10^{1}$	5.1214$\;\times 10^{1}$	5.1108$\;\times 10^{1}$	5.1214$\;\times 10^{1}$	5.2769$\;\times 10^{-1}$
	ARO	4.9154$\;\times 10^{1}$	5.1214$\;\times 10^{1}$	5.0567$\;\times 10^{1}$	5.0761$\;\times 10^{1}$	6.6831$\;\times 10^{-1}$
	DO	4.6423$\;\times 10^{1}$	5.1214$\;\times 10^{1}$	5.0095$\;\times 10^{1}$	5.1214$\;\times 10^{1}$	1.6393$\;\times 10^{0}$
	FOX	4.9877$\;\times 10^{1}$	5.1214$\;\times 10^{1}$	5.0886$\;\times 10^{1}$	5.0915$\;\times 10^{1}$	3.7241$\;\times 10^{-1}$
	JS	4.6545$\;\times 10^{1}$	5.1214$\;\times 10^{1}$	4.8294$\;\times 10^{1}$	4.7234$\;\times 10^{1}$	1.8209$\;\times 10^{0}$
	SCA	5.0181$\;\times 10^{1}$	5.1214$\;\times 10^{1}$	5.1066$\;\times 10^{1}$	5.1214$\;\times 10^{1}$	**2.7425** $\;\times 10^{-1}$
	TSA	5.0131$\;\times 10^{1}$	5.1213$\;\times 10^{1}$	5.0694$\;\times 10^{1}$	5.0649$\;\times 10^{1}$	3.1005$\;\times 10^{-1}$
	**WOA**	4.4948$\;\times 10^{1}$	**4.9481** $\;\times 10^{1}$	4.6931$\;\times 10^{1}$	4.6970$\;\times 10^{1}$	1.0908$\;\times 10^{0}$
	**EJSARO**	**3.0066** $\;\times 10^{1}$	5.1214$\;\times 10^{1}$	**3.7794** $\;\times 10^{1}$	**3.4869** $\;\times 10^{1}$	8.1023$\;\times 10^{0}$

**Table 8:** The RI values of the EJSARO over the competitors.

Network	EJSARO vs.								
	AEO	AO	ARO	DO	FOX	JS	SCA	TSA	WOA
1	36.04	39.26	40.24	42.11	52.48	39.78	46.24	47.00	41.14
2	34.48	37.08	42.32	46.45	54.68	39.01	48.92	49.10	42.31
3	32.13	28.11	39.78	42.60	54.21	35.06	44.85	47.81	35.82
4	29.23	23.98	37.47	45.33	57.86	37.50	42.80	50.94	37.40
5	44.10	47.50	51.04	51.44	59.94	48.35	55.72	57.13	49.41
6	30.53	28.13	37.47	41.54	50.82	34.18	46.05	45.75	31.35
7	24.72	27.47	31.76	34.78	41.68	28.20	39.16	37.21	32.93
8	18.71	17.89	27.27	31.62	37.14	23.57	31.86	34.35	19.96
9	31.74	26.98	41.32	44.81	53.73	35.61	45.79	46.77	35.64
10	26.94	22.22	36.75	40.20	49.25	30.82	41.70	42.08	32.53
11	27.22	26.86	35.69	39.91	50.14	32.82	38.60	43.31	34.91
12	28.79	31.94	38.01	40.60	48.89	34.88	43.00	44.99	34.41
avg.	30.39	29.78	38.26	41.78	50.90	34.98	43.72	45.54	35.65

## Data Availability

The original contributions presented in this study are included in the article. Further inquiries can be directed to the corresponding author.

## Appendix A

The appendix compares the existing metaheuristic-based localization methods explored in Section 2. The comparison is presented in Table A1.

**Table A1:** A brief comparison of existing metaheuristic-based positioning/localization methods.

Ref.	Optimizer	Positioner	Env.	Evaluation Metrics	Strengths	Weak Points	Year
[18]	GWO	DV-Hop	2D IoT	Normalized average localization error	Dual-radius broadcasts High performance with low anchors Range-free and simple to deploy Comprehensive refinement of hop count Dynamic weights and adaptive updates Energy-aware design	Higher computational load More complex than DV-Hop due to IGWO Parameter sensitivity Ignore real-world issues like signal noise or obstacles. More control traffic in large deployments due to dual-radius strategy	2025
[19]	BKA	DV-Hop	2D WSN	Everage localization error Running time Convergence rate	Stable convergence Error-corrected distance model Robust search strategy Range-free and low-cost	Slower than DV-Hop Anchor-density sensitive Hop propagation relies on network-wide broadcasts Parameter tuning required	2025
[11]	GOA	DV-Hop	2D WSN	Localization error Runtime Convergence rate	Rapid refinement of estimated positions Low runtime No specialized hardware is needed Effective in less GPS-enabled nodes Adjustable parameters	Not suitable for low-power applications Dependency on initial DV-Hop estimates Requires careful tuning of parameters Converge to local optima, especially in irregular topologies	2024
[20]	RSA	LLE	2D IoT	Localization error Energy consumption Time	Noise resistance Scalability to large-scale networks Suitable for dense IoT environments. Lower energy consumption Dimensionality reduction for complex data	No real-world experiments High computational cost Parameter sensitivity Limited comparative evaluation Static topology assumption	2024
[4]	SMA EO	RSSI	3D IoT	Localization Mean square error Wilcoxon signed-rank test	Adaptive learning mechanism Low variance Fast convergence Suitable for 3D environments	High computational complexity Sensitivity to RSSI quality Many tunable parameters Single-source data (RSSI-only)	2023
[5]	TSA HHO	DV-Hop	2D IoT	Average localization error Node localization error Localization error variance	Being range free Do not need distance/range measurements Suitable for harsh environments, Optimized anchor placement Balanced exploration and exploitation	High computational complexity needs more resources to processing of anchor-node No experiments on noisy or dynamic environments Dependency of the performance on network topology and anchor density	2022
[21]	GWO	Clock asynchronous localization system	3D UWSN	Location accuracy Location coverage	Lower energy consumption Do not reliance on active nodes Higher coverage Motion prediction Balanced balance and exploration	High computational complexity High computational load Performance is not validated in harsh environments Dependency on node behavior Sensitive parameters	
[22]	COA SOA	-	2D WSN	Localization error Energy consumption	Using mobile sink and multi-hop routing Low energy consumption Balanced network load Flexible in dynamic network topologies	High algorithmic overhead environmental effects are not tested Predictable motion of sink Require parameter tuning for different scenarios	2023
[23]	PSO TS	RSSI	2D WSN	Localization accuracy Convergence	High accuracy Hybrid optimization Fast Convergence Constraint-guided search Realistic signal modeling	RSSI Sensitivity High complexity for constraint layers Trilateration dependency Simulation-based validation Not suitable for limited anchors or signal obstruction	2023
[24]	CSA PSO	TDOA AOA	NLoS	Node positioning accuracy Convergence rate	High localization precision High convergence and adaptability Noise resistance Do not need additional expensive sensors Better swarm management	High processing overhead Dependency on parameter tuning Idealized Simulations Static environment assumption Inapplicable in high-mobility scenarios	2023
